# Deciphering the Genetic Programme Triggering Timely and Spatially-Regulated Chitin Deposition

**DOI:** 10.1371/journal.pgen.1004939

**Published:** 2015-01-24

**Authors:** Bernard Moussian, Annalisa Letizia, Guillermo Martínez-Corrales, Bárbara Rotstein, Andreu Casali, Marta Llimargas

**Affiliations:** 1 Institut de Biologia Molecular de Barcelona, CSIC, Barcelona, Spain; 2 Animal Genetics, Interfaculty Institute for Cell Biology, University of Tuebingen, Tuebingen, Germany; 3 Institute for Research in Biomedicine (IRB Barcelona), Barcelona, Spain; Harvard Medical School, Howard Hughes Medical Institute, UNITED STATES

## Abstract

Organ and tissue formation requires a finely tuned temporal and spatial regulation of differentiation programmes. This is necessary to balance sufficient plasticity to undergo morphogenesis with the acquisition of the mature traits needed for physiological activity. Here we addressed this issue by analysing the deposition of the chitinous extracellular matrix of *Drosophila*, an essential element of the cuticle (skin) and respiratory system (tracheae) in this insect. Chitin deposition requires the activity of the chitin synthase Krotzkopf verkehrt (Kkv). Our data demonstrate that this process equally requires the activity of two other genes, namely *expansion* (*exp*) and *rebuf* (*reb*). We found that Exp and Reb have interchangeable functions, and in their absence no chitin is produced, in spite of the presence of Kkv. Conversely, when Kkv and Exp/Reb are co-expressed in the ectoderm, they promote chitin deposition, even in tissues normally devoid of this polysaccharide. Therefore, our results indicate that both functions are not only required but also sufficient to trigger chitin accumulation. We show that this mechanism is highly regulated in time and space, ensuring chitin accumulation in the correct tissues and developmental stages. Accordingly, we observed that unregulated chitin deposition disturbs morphogenesis, thus highlighting the need for tight regulation of this process. In summary, here we identify the genetic programme that triggers the timely and spatially regulated deposition of chitin and thus provide new insights into the extracellular matrix maturation required for physiological activity.

## Author Summary

In this work we studied the maturation of the extracellular matrix during *Drosophila* embryogenesis. *Drosophila* deposit a chitin-rich extracellular matrix with key physiological functions, such as the control of organ size and shape, and cuticle formation. Chitin synthesis depends on chitin synthases, and in *Drosophila* the gene *krotzkopf verkehrt* (*kkv*) encodes the main enzyme of this family. Our observations indicate that Kkv alone is not sufficient to induce chitin formation. We have identified another function (which is exerted by the activity of two genes encoding MH2-domain proteins) that are equally required for chitin deposition. The most striking result of our analysis is that the presence of Kkv and the newly identified function is sufficient to trigger chitin deposition in ectodermally-derived tissues, even if they are normally devoid of this polysaccharide. Importantly, we also demonstrate that unregulated chitin deposition (absent, advanced, or ectopic) leads to severe defects in morphogenesis. We show that the temporal and spatial pattern of *kkv* and the other two genes perfectly recapitulates the deposition of chitin, thereby unveiling a highly co-ordinated mechanism for the acquisition of mature traits.

## Introduction

Organ formation requires a finely tuned temporal and spatial control of events. Once cells have acquired the organ cell fate, they undergo a series of consecutive morphogenetic steps until they reach the mature and physiological state, which is then maintained by homeostasis. Many examples in the literature illustrate the failure of organ formation when cells cannot reach their final differentiated state. However, the premature acquisition of mature traits may also lead to deleterious effects.

A general feature of the maturation of many organs and tissues is the deposition of an extracellular matrix (ECM). The ECM provides biochemical and structural support, participates in cell adhesion, segregates and protects tissues, regulates cell-cell communication, and senses and transduces mechanical signals [1,2]. Insect epithelial cells (in particular epidermal, tracheal, foregut, and hindgut cells) deposit a specialised ECM at the end of embryogenesis known as the cuticle, which is renewed during moulting and metamorphosis. The cuticle serves as an exoskeleton and provides protection against dehydration, predators, and pathogens [3]. A major component of the cuticle is the polysaccharide chitin, a polymer of UDP-N-acetylglucosamine (GlcNAc) monomers synthesised by the Leloir pathway [4,5,6,7,8,9]. Chitin is deposited in a highly organised arrangement at the apical surface of epidermal and tracheal cells to form the cuticle [10]. Independently, and before the deposition of this apical tracheal cuticle, a matrix that contains a chitin filament and chitin-binding proteins assembles transiently inside the lumen of the tracheal tubes in *Drosophila melanogaster*. This chitinous matrix plays a key role in the regulation of tracheal tube size and shape [5,11,12,13,14,15,16]. Chitin is produced by glycosyltransferase chitin synthases (CHS), which polymerise the GlcNAc monomers, thus forming linear polymers [17,18]. CHS reside in the apical membrane and form a pore through which the nascent polymerised chitin fibers are extruded. However, the exact mechanism by which CHS polymerise and extrude chitin is not fully understood.

Here we report the mechanism involved in the timely and spatially regulated chitin deposition in *Drosophila*. Our results demonstrate that chitin deposition requires two functions, one exerted by the already known class A chitin synthase Krotzkopf verkehrt (Kkv) and a second by two MH2-containing proteins, Expansion and Rebuf (Exp and Reb). Exp/Reb perform the same function and are an absolute requirement for chitin deposition. In their absence, the luminal chitin filament is not assembled and the tracheal and epidermal cuticles are chitin-less, an identical phenotype to that of *kkv* mutants. In agreement with the absolute requirement of both functions, we found that the pattern of expression of these genes fully accounts for the regulated chitin deposition. When *exp/reb* genes are over- or misexpressed, they bring about early and increased chitin deposition in places where *kkv* is normally expressed. Strikingly, the simultaneous misexpression of *kkv* and *exp/reb* promotes chitin deposition in ectopic ectodermally-derived tissues. This observation demonstrates that together both activities are not only required but are also sufficient to promote chitin deposition. Our analysis shows that unregulated chitin deposition impairs morphogenesis, thus highlighting the need of a finely tuned control of deposition. At the cellular level, we found that Exp/Reb accumulate strongly at the apical membrane, colocalising with Kkv in an independent manner, and that this subcellular localisation correlates with chitin deposition. Our results suggest that Exp/Reb could be involved in the translocation of the Kkv-synthesized chitin polymers across the membrane and/or their release into the extracellular domain to form microfibrils. In summary, here we unveil a highly regulated developmental mechanism that exquisitely ensures the coordinated acquisition of a mature trait during organ formation. Furthermore, we provide a clear case in which the premature acquisition of a mature trait leads to morphogenetic defects. Finally, our results may also provide new targets for the control of insect plagues through the regulation of chitin deposition, as putative orthologs of these genes are found in the ecdysozoa clade.

## Results

### CG13188 and CG13183 encode MH2-containing proteins expressed in the tracheal system

**Figure 1:**
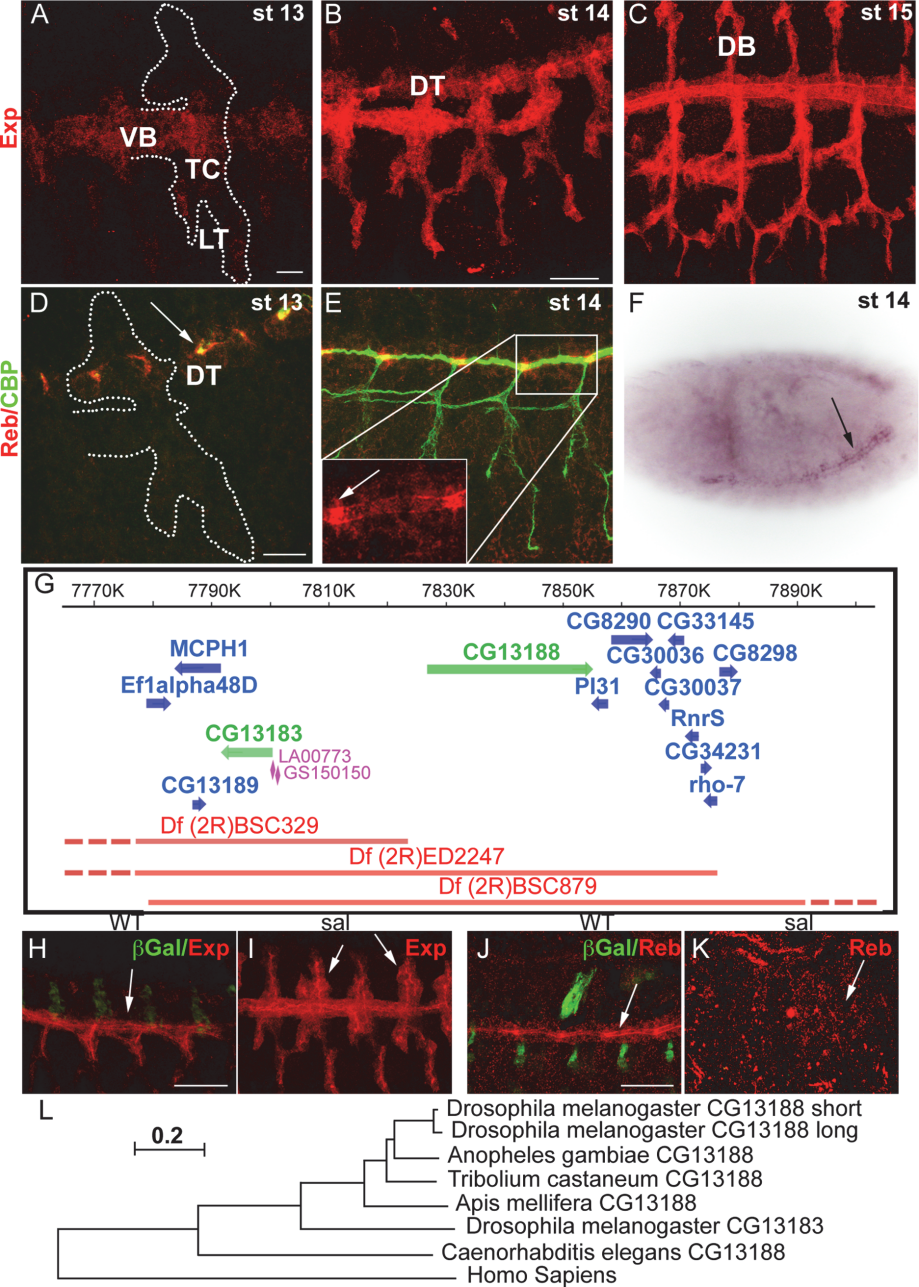
Tracheal expression of *exp* and *reb*. (A-E, H-K) Projections of confocal sections of embryos at the indicated stages. (F) Bright field image of a whole mount ISH in a dorsolateral view. (A-C) Exp is expressed in the tracheal system in a dynamic pattern, being first present in the VB, LT and TC and later in the DT and DB. (D-F) *reb* is expressed only in the DT region (arrow in F). The protein localises mainly apically and more strongly in the branch fusion region (arrowheads in D,E). (G) Scheme of the genomic region and of the deficiencies and transgenes used. The genes uncovered by the deficiencies are indicated. (H-K) Sal negatively regulates *exp* expression, as in its absence Exp is expressed in the DT-DB region already at stage 13 (arrowheads in I). In contrast, in *sal* mutants, Reb is not expressed (arrows in J,K). (L) Evolutionary tree of CG13188 (short and long isoforms) and CG13183 obtained using the MEGA 5.2.2 software from a Clustal O-alignment of homologous sequences from insects and non-insects (shown in S1 Text). The human Smad protein “Mothers against Dpp homolog 3 (isoform X1)”, which also has an N-terminal MH2 domain, is the closest CG13183-similar human protein and was included as an out-group. The *Apis mellifera* and *Tribolium castaneum* sequences were added to the alignment as examples of expectedly distant orthologues [43]. The sequence of the dipteran *Anopheles gambiae* was included as an example of an expectedly close orthologue. The scale bar in the figure indicates changes per site (residue), thereby implying the evolutionary distance. Scale bars A,D 10 μm, B,H,J 25 μm.

In the course of a microarray analysis, we identified CG13188 (named *expansion* (*exp*) in a recent independent publication [19]) as a target of Ttk [20]. BDGP reported expression of this gene in the tracheal system and in the epidermis at late embryonic stages. We raised an antibody against the protein isoform B, which is the one expressed in the embryo [19]. Antibody stainings confirmed the expression and allowed us to refine the temporal pattern in the trachea: Exp protein was first detected at late stage 12-early stage 13 in the Visceral Branch (VB), Transverse Connective (TC), and Lateral Trunk (LT) region (Fig. 1A). This pattern extended first to the Dorsal Trunk (DT) (Fig. 1B) and later to the Dorsal Branches (DBs) (Fig. 1C) during stage 14–15. A search for similar genes identified the gene CG13183 (*rebuf, reb*) (56% aa similarity), which lies next to CG13188 (Fig. 1G). BDGP reported the expression of *reb* in the tracheal DT, and our *in situ* hybridisation experiments confirmed this pattern (Fig. 1F). We also raised antibodies against Reb, which confirmed expression exclusively in the DT from early stage 13, with a stronger accumulation in the DT fusion region (Fig. 1D,E).

The spatiotemporal tracheal pattern of Exp and Reb indicated branch-specific regulation. We found that the transcription factor Spalt (Sal), which is first restricted to the dorsal part of the trachea and later to the DT [21], negatively regulates the initial pattern of Exp in the dorsal part (Fig. 1H,I). In contrast, Sal positively regulates Reb expression in the DT (Fig. 1J,K).

The molecular analysis of Exp and Reb proteins identified a single recognisable SMAD/FHA domain (also called MH2). MH2 domains are typically found in members of the Smad family [22,23], which mediate the TGFβ signal, thus raising the possibility that these two genes participate in the TGFβ pathway. However, our functional characterisation showed that Exp and Reb do not transduce the TGFβ signal in the trachea but perform a different activity (see below and Beich-Frandsen et al. in preparation). A similar conclusion has recently been published [19].

Homology searches with CG13188 and CG13183 revealed the presence of orthologous sequences only in invertebrates, including arthropods and nematodes. No homologous sequence was found in fungi that also produce extracellular chitin. A subset of the retrieved sequences from insects and non-insects was used to generate an evolutionary tree (Fig. 1L, S1 Text; Beich-Frandsen under review for further details). The data suggested that CG13188 represents the insect ancestral protein in *Drosophila*. In Drosophilids but not other Dipterans like *Anopheles gambiae*, CG13188 duplicated to give rise to CG13183. Orthologs of CG13188 were found in distant species like *Apis mellifera* (honeybee) and *Tribolium castaneum* (red flour beetle). Interestingly, treatment of *T. castaneum* larvae or pupae with dsRNA against the orthologue *TC010825* causes lethality (http://ibeetle-base.uni-goettingen.de/details/iB_01740), indicating the functional requirement of the gene.

### *exp* is required for luminal chitin deposition in the trachea

**Figure 2:**
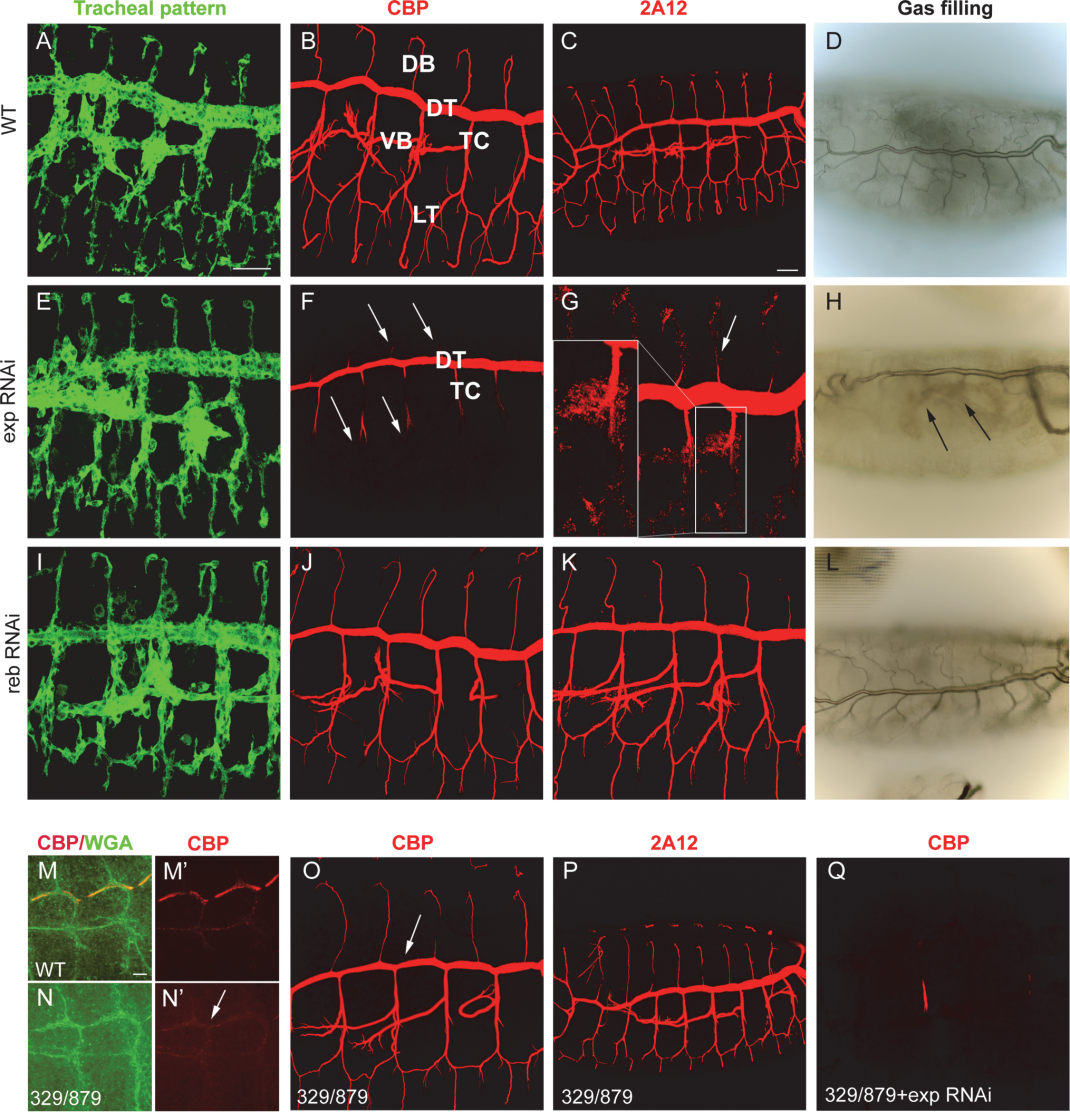
Tracheal defects in *exp* and *reb* loss-of-function. All images are projections of confocal sections except D, H, and L, which are bright field images. All images show tracheal metameres of embryos at stage 13 (M,N), larval stage (D,H,L), or stage 15 (rest of panels). In WT embryos (A), chitin (B) and chitin-associated proteins (C) accumulate in the lumen of all tracheal branches, and by the end of embryogenesis the trachea fill with air (D). The down-regulation of *reb* does not generate detectable defects (I-L). In contrast, *exp* down-regulation prevents luminal accumulation of chitin and associated proteins in all branches (arrows in F,G), except the DT and part of TC, while the pattern of branching is normal (E). Later, only the DT is filled with air (arrows in H). In the absence of *reb*, chitin deposition in the DT is delayed (arrow in N’, compare to M’). Here WGA allows visualisation of the apical region of the trachea (M,N). However, at later stages, chitin (O) and associated proteins (P) are present (with slightly lower DT levels, arrow in O). When *exp* is down-regulated in the deficiency combination, no chitin accumulates in the trachea (Q). Scale bars A,C 25 μm, M 7.5 μm.

We tested the requirements for *exp* by expressing RNAi lines in the trachea. Tracheal down-regulation of the gene (around 70% decrease by qPCR, S1A-C Fig.) produced no detectable defects in the pattern of migration (Fig. 2A, E), organisation (S1D-E Fig.), or diversification of tracheal cells (S1F-G Fig.). However, we detected a clear defect in chitin deposition when we used a marker for chitin (chitin binding probe, CBP). In the wild type, a chitin filament is deposited transiently inside the lumen during the tube expansion period [15] (Fig. 2B). *exp* tracheal down-regulation prevented luminal chitin accumulation in dorsal and ventral branches (i.e. the DB, LT and VB), remaining only in the DT and part of the TC (Fig. 2F, compare to 2B). In the wild type, several proteins, such as Gasp (visualised with 2A12) and Vermiform (Verm) [12,16,24], accumulate in the lumen with the chitinous matrix (Fig. 2C, S1H). In animals with reduced *exp* function, these proteins did not accumulate in the lumen of dorsal and ventral branches, but instead remained in the cytoplasm (Fig. 2G, S1I), further indicating that the chitin filament was not properly formed. In the wild type, tracheal branches become physiologically functional and fill with gas at the end of embryogenesis (Fig. 2D). Only the branches that accumulated chitin (i.e. DT) filled with gas in *exp* down-regulation, possibly causing larval death by asphyxia. We also noted the presence of apical expansions (S1J-K Fig.), as recently reported [19].

### The role of *reb* in luminal chitin deposition in the trachea

Using RNAi, we tested the tracheal requirement for *reb*. RNAi expression produced no detectable defects in the pattern, migration, or diversification of tracheal cells, or in gas filling (Fig. 2I-L and not shown). Nor did RNAi prevent chitin accumulation in the trachea (Fig. 2J). We tested the effects of the absence of *reb* by using a combination of deficiencies (BSC329/BSC879) that removes *reb* and three other genes (Fig. 1G), excluding *exp*. Mild defects in chitin deposition were detected. We observed that in the wild type, chitin deposition starts in the DT region at stage 13. By early stage 14, deposition expands first to the VB and then to the TC and LT. From late stage 14, chitin accumulates in all the branches, including the DB, and very strongly in the DT (S2A-H Fig.). In the mutants, chitin accumulation in the DT at stage 13 was delayed (Fig. 2M,N), and later chitin levels in the DT were slightly lower than in the wild type (Fig. 2O-P). This result indicates that *reb* is involved in chitin deposition.

The mild defects in chitin deposition in the absence of *reb* could be due to the presence of *exp*, which is expressed in all tracheal cells, including the DT. To test this hypothesis, we down-regulated *exp* in the absence of *reb*. Embryos showed a normal branching pattern (S2I Fig.) but were devoid of the chitin filament (Fig. 2Q, S2I’). Branches did not fill with air at the end of embryogenesis, and the embryos died (S2J Fig.). For this reason, we named the gene *rebuf*, which in Catalan means “huff and buff”.

These results show that *reb* is also required for chitin deposition in the DT. When *reb* is absent, chitin is still deposited in this trunk due to the presence of *exp*. Similarly, when *exp* is down-regulated, chitin is deposited in the DT as a result of the presence of *reb*. Thus, *reb* is largely dispensable when *exp* is present but is an absolute requirement for luminal tracheal chitin deposition in the absence of *exp*. All together, these observations indicate that *exp* and *reb* exert redundant functions on chitin deposition.

### *exp* and *reb* are an absolute requirement for general chitin deposition and cuticle formation

**Figure 3:**
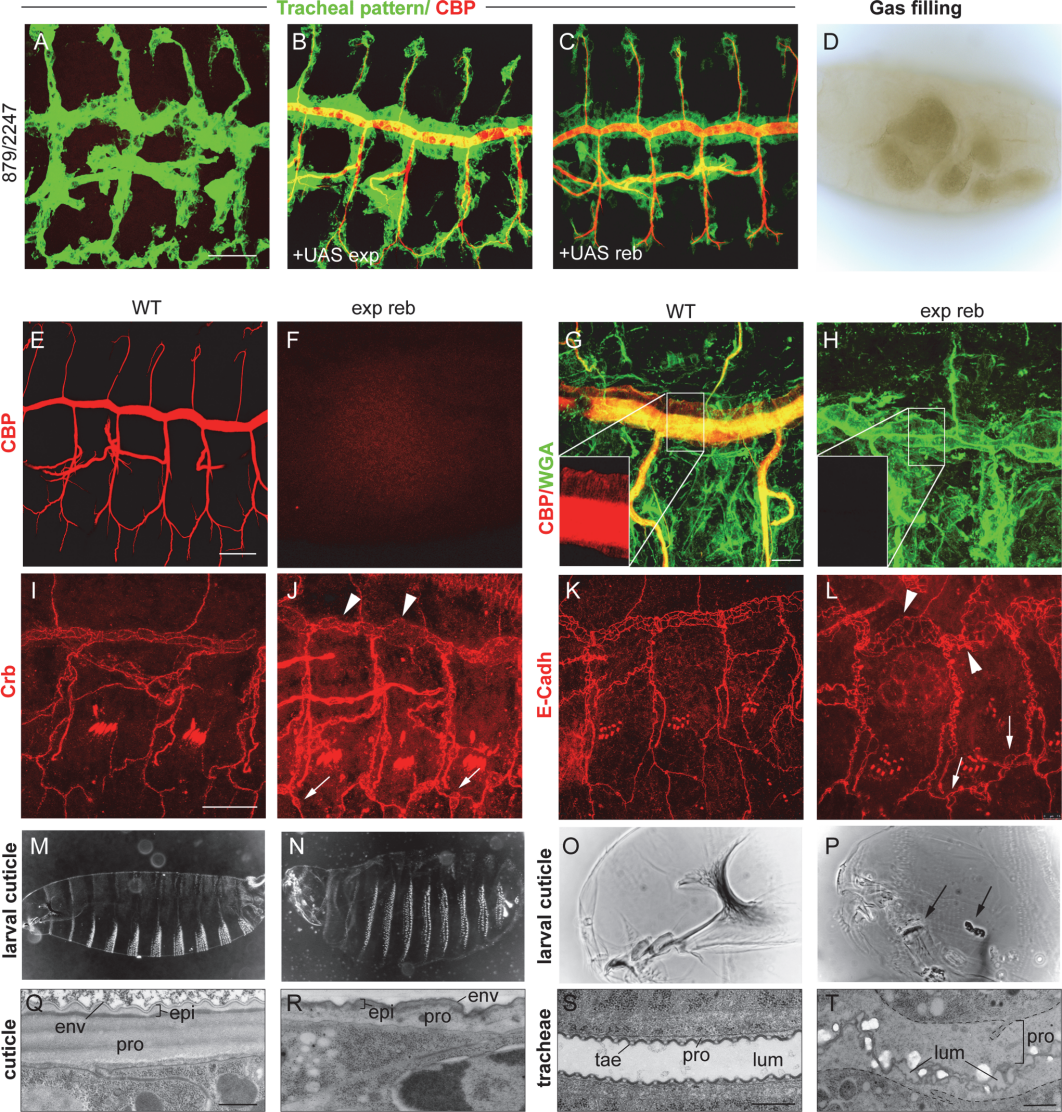
Phenotypes of *exp reb* mutants. (A-C and E-L) Projections of confocal sections of embryos at stage 14 to 16. (D,O,P) bright field and (M,N) dark field images of early larvae. (Q-T) TEM micrographs. Lack of tracheal chitin filament in deficiency combination mutants (A) can be rescued by adding back either *exp* (B) or *reb* (C) in the trachea. The absence of *exp* and *reb* prevents luminal (F) and cuticular (H) chitin deposition. Apical markers show a normal apicobasal polarity and adhesion, but also the presence of dilations and expansions in the DT (arrowheads in J,L) and apical expansions in lateral branches (arrowheads in J,L). Mutants show inflated larval cuticles (N) and defects in the mouth cuticular structures (arrows in P). At the ultrastructural level, the wild-type mature cuticle (Q) consists of three composite layers: envelope (env), epicuticle (epi), and the inner procuticle (pro, filled with fibrous chitin). In *exp reb* mutants (R) the envelope and epicuticle are thin, and the procuticle is devoid of any fibrous structure and is thinner than that of the wild-type. In the tracheal cuticle, the lumen (lum) is stabilised by the spiral cuticle. The ridges of the cuticle are the taenidia (tae), formed by a thick procuticle (pro), a thin and electron-dense epicuticle, and the envelope (S). The cuticle of *exp reb* mutants (T) detaches from the surface of tracheal cells. The procuticle is bloated and contains little material. The lumen collapses. Scale bars, A,E,I 25 μm, G, 10 μm, Q,S,T 500 nm.

We next studied the effects of the absence of both genes. For this purpose, we used a combination of two deficiencies (BSC879/ED2247) that uncover *exp* and *reb* and 12 other genes (Fig. 1G). The transheterozygous embryos did not accumulate the tracheal chitin filament (Fig. 3A, F), and the trachea remained uninflated (Fig. 3D). Chitin filament formation was fully rescued by adding back either *exp* or *reb* in the tracheal cells (Fig. 3B, C), thereby indicating that the defects are caused exclusively by the absence of these genes. Thus, we used this deficiency combination as a null condition for both *exp* and *reb* (hereafter *exp reb* mutants).

We examined the tracheal defects of *exp reb* mutants, observing a normal pattern of migration and tracheal cell specification (Fig. 3A); however, branches completely lacked luminal and apical-cuticular chitin (Fig. 3A, E-H). Staining with the apical determinant Crumbs (Crb) and the Adherens Junctions marker E-Cadherin (E-Cadh) indicated that the general apicobasal polarity and adhesion, respectively, were not affected (Fig. 3I-L). However, these and other apical markers (S4A-C Fig.) also revealed a cystic appearance of the DT, with many dilations and constrictions, and the presence of apical expansions, usually at the joints between branches. E-Cadh stainings also revealed abnormal cell shapes in the DT.

In addition to the trachea, chitin is also deposited in the procuticle layer of the epidermal cuticle. Exp is expressed in the epidermis at late embryonic stages (http://insitu.fruitfly.org), thus raising the possibility that it is also required for epidermal chitin deposition. To test this, we down-regulated *exp* by RNAi using widely expressed Gal4 drivers (*69B* or *tubulinGal4, tubGal4*). We detected a clear phenotype attributable to chitin defects: the embryos appeared inflated and were shorter (probably due to the inflation) (S1L-M Fig.), and showed defects in the head skeleton. Occasionally, we detected a defect of mouth inversion or rotation (in 20% of embryos, n = 20) (S1N-O Fig.). These defects were more severe in the absence of *exp* and *reb*, where we observed a fragmented and granular head skeleton and an inflated and misshapen larval shape (Fig. 3M-P). The defects observed were identical to those of *kkv* mutants (see following chapters).

To further demonstrate a chitin deposition defect, we examined cuticle deposition by transmission electron microscopy (TEM). The tracheal cuticle forms a spiral structure that constitutes the inner wall of the tube. It is composed of a chitinous procuticle, a thin chitin-less epicuticle, and an envelope that covers the whole structure. A thicker procuticle accounts for the spiral ridges, the so-called taenidiae. In *exp reb* mutants, the tracheal envelope and epicuticle detached from the epithelial cells, while the space between these structures and the epithelial cells were devoid of material (Fig. 3S, T). This phenotype is indistinguishable from the phenotype of *kkv* mutants [25]. We also studied the cuticle of the integument. The wild-type cuticle consists of three histologically distinct horizontal layers: the outermost envelope, the middle epicuticle, and the innermost procuticle (Fig. 3Q). Chitin microfilaments are arranged in parallel sheets (laminae) that are stacked helicoidally within the procuticle, resulting in a crystalline organisation of chitin. The envelope and the epicuticle of *exp reb* mutants showed a decrease in thickness. In addition, the procuticle in *exp reb* mutants was amorph (Fig. 3R), resembling the procuticle of *kkv* mutants (see below), which do not deposit chitin [25,26].

Taken together, our results show that *exp* and *reb* are required for general chitin deposition in the embryo, which is absolutely required for the tracheal luminal filament and cuticle organisation.

### *reb* overexpression brings about early and increased chitin deposition, thus affecting tracheal morphogenesis

**Figure 4:**
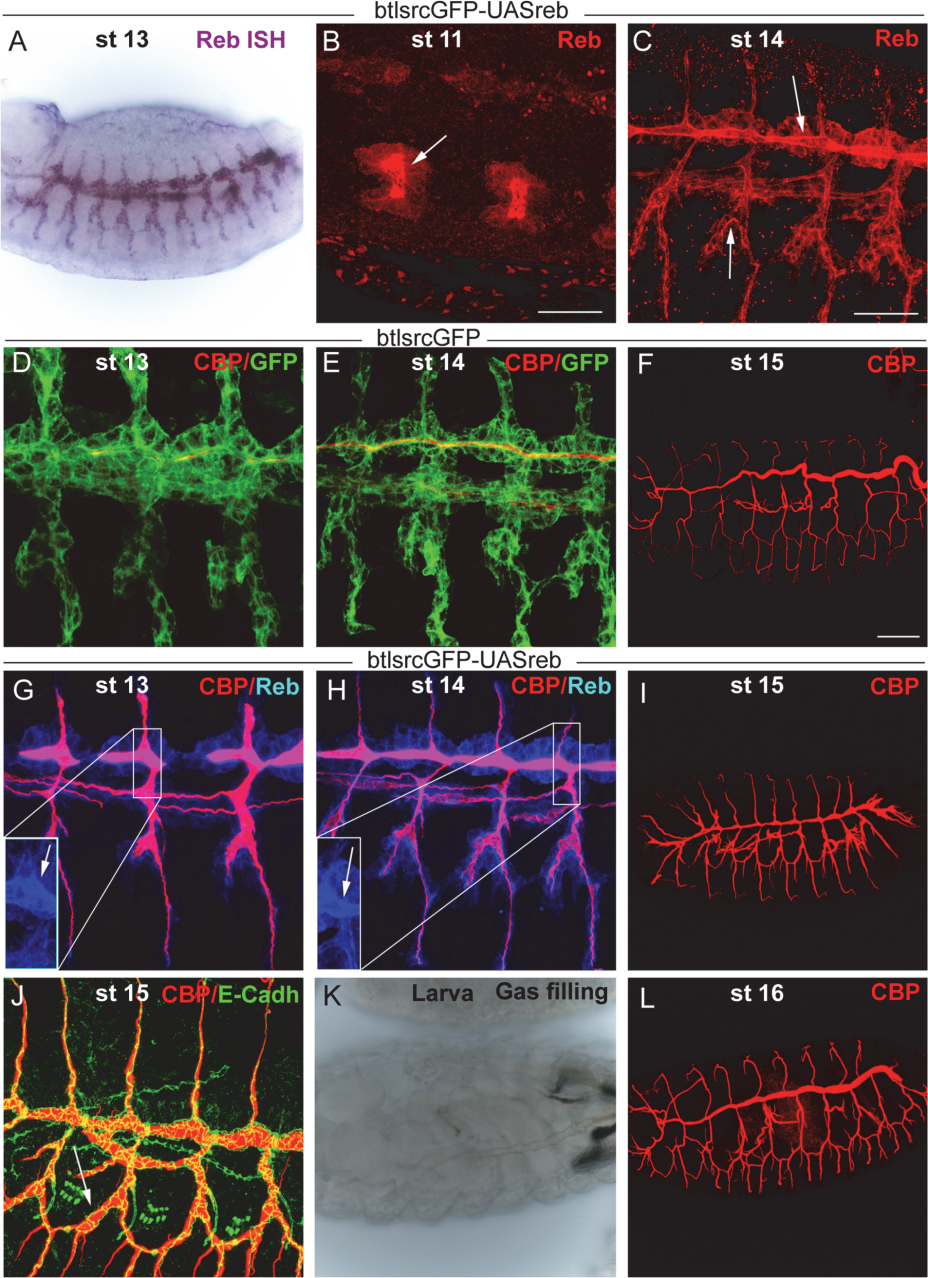
Effects of *reb* tracheal overexpression. (B-J and L) Projections of confocal sections of embryos at the indicated stages. (A,K) Bright field images. Tracheal *reb* overexpression (using LA00733) leads to a strong accumulation of the protein, mainly apically (arrowheads in B,C,G,H). It also brings about early and increased chitin deposition (G,H). Consequently, tracheal morphogenesis in terms of branching (I) and intercalation (J) is affected, and the larval tracheal system does not inflate (K). These defects are reverted when *reb* is down-regulated (L). Scale bars B,C 25 μm, F 50 μm.

We addressed whether the restricted expression of *reb* was functionally relevant by performing over- and mis-expression experiments. Tracheal expression of GS15050 and LA00773 (two independent P-UAS lines inserted in front of *reb*, Fig. 1G) produced a high overexpression of the gene in all tracheal cells from early stage 11 (Fig. 4A-C). This expression produced clear effects on the deposition of chitin, as it started to accumulate strongly at early stage 13 not only in the DT but in all tracheal branches (Fig. 4D, G). This generalised increase in chitin accumulation was maintained throughout development (Fig. 4H,I). By the end of embryogenesis, we observed a fully penetrant strong phenotype: branches were straight and apparently shrank (particularly the DT) (Fig. 4F, I), and the LT displayed lack of cell intercalation (Fig. 4J), as cells remained connected by intercellular junctions [27]. In addition, the trachea did not fill with air, indicating that it was not physiologically functional (Fig. 4K). We attribute these morphogenetic defects to the premature and excessive chitin deposition. This phenotype was completely rescued by simultaneous expression of a *reb*RNAi construct (Fig. 4L) (but not by overexpression of other UAS-lines, S3E-F Fig.). This result validates *reb*RNAi as a functional line and indicates that the defects produced by GS15050/LA00773 tracheal expression are due exclusively to the overexpression of *reb*. The overexpression of *exp* produced similar, although milder defects (S3A-D Fig.).

Taken together, these results show that Reb overexpression brings about advanced and increased chitin deposition and that this leads to defects in tracheal tube morphogenesis. These findings thus highlight the relevance of the tight regulation of *reb*.

### *exp reb* phenotypes are identical to those of *kkv*

**Figure 5:**
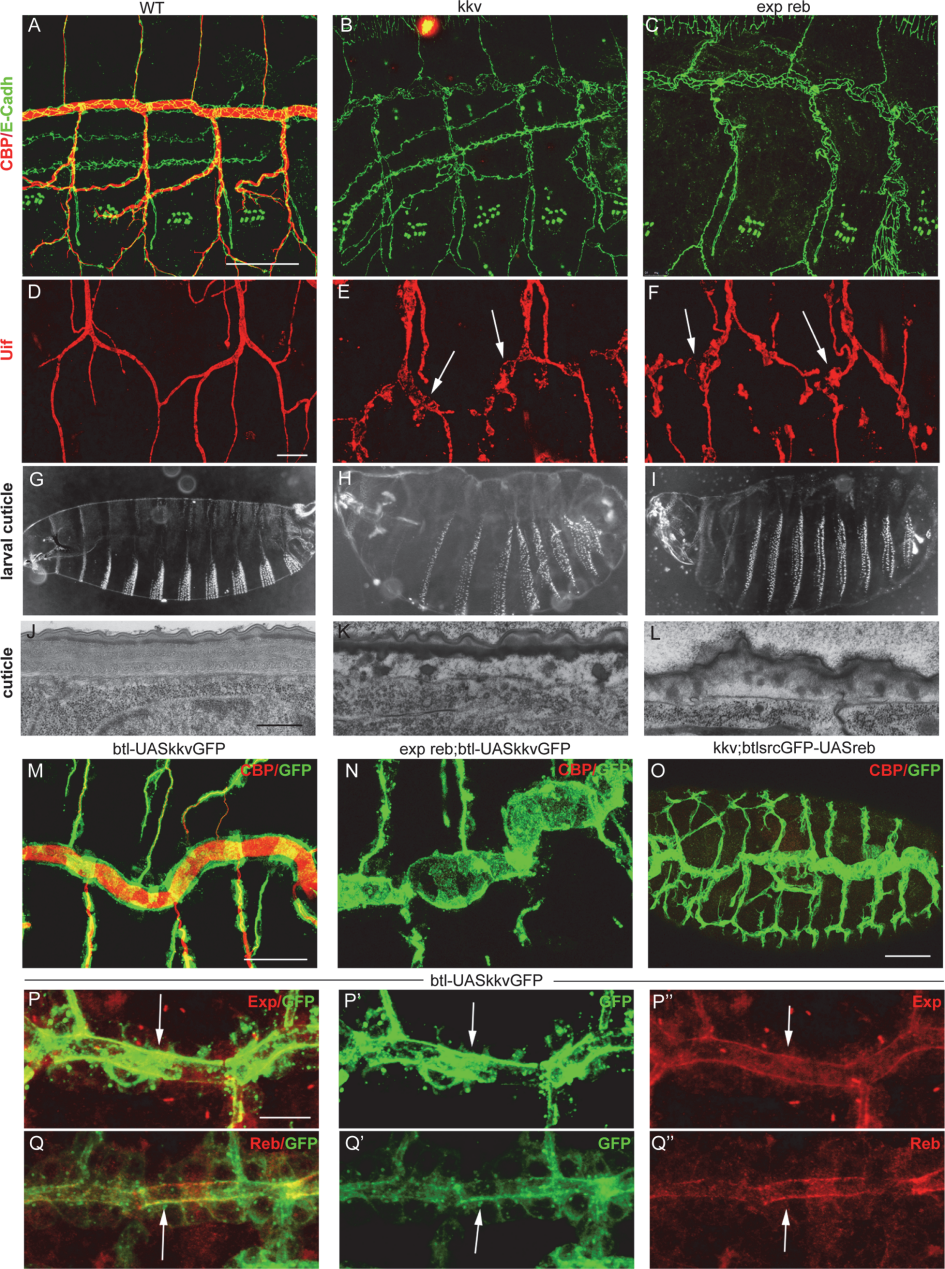
Analysis of *exp reb* and *kkv* phenotypes, epistatic interactions, and subcellular accumulation. (A-F and M-Q) Projections of confocal sections of embryos at stage 15. (G-I ) Dark field images. (J-L) TEM micrographs. (A-I) Note the similar tracheal defects of *kkv* and *exp reb* mutants. (J-L) In *exp reb* and *kkv* mutant embryos the procuticle is amorph and thin. Probably as a consequence, the envelope and the epicuticle appear to be disorganised. (M-O) Epistatic experiments show that *kkv* overexpression does not rescue the absence of *exp/reb* and *reb* does not rescue *kkv* defects. (P-Q) Analysis of subcellular accumulations shows localisation of KkvGFP (arrow in P’,Q’), Exp (arrow in P’’) and Reb (arrow in Q’) in the apical membrane of tracheal cells. There, we detect a partial colocalisation of KkvGFP and Exp (arrow in P) or Reb (arrow in Q). Scale bars A,M 25 μm, D 10 μm, P 7.5 μm, J 500 nm.

*kkv* encodes the chitin synthase required for luminal and cuticular chitin deposition in the trachea and epidermis [5,15,25]. We therefore compared the phenotypes of *kkv* and *exp reb* mutants. The absence of *kkv* produced similar defects to those caused by the depletion of Exp/Reb. In particular, in *kkv* and *exp reb* mutants the cuticular and luminal chitin of the tracheae was absent (Fig. 5A-C) and there were dilations and constrictions in the DT and apical expansions in other branches (Fig. 5D-F, S4A-C). Both mutants displayed defects in the luminal accumulation of several markers, such as Gasp, ANFGFP (used as a secretion marker [28]) or Verm (S4D-L Fig.), but not of other markers like Pio-pio (S4M-O Fig.). In addition, the defects in cuticle formation were comparable in both mutants (Fig 5G-L, S4P-R). These similarities raised the possibility that these genes act in the same genetic pathway required for chitin deposition.

To test this hypothesis, we performed epistatic experiments. The tracheal expression of *kkvGFP* rescued the defects of a *kkv* mutant and restored the luminal accumulation of chitin (S4S Fig.). However, this protein was unable to restore chitin accumulation produced by the absence of *exp reb* or by the down-regulation of *exp* (Fig. 5M, N, S4T). On the other hand, while *exp* and *reb* rescued luminal chitin in *exp reb* mutants (Fig. 3B, C), they did not restore chitin accumulation in a *kkv* mutant background (Fig. 5O). These results indicate that *kkv* is not required for *exp/reb* expression and that *exp/reb* are not required for *kkv* expression. But most importantly, these epistatic experiments also showed that both functions are required in parallel for chitin deposition and that the presence of one cannot substitute the other when this is absent.

### Exp/Reb and Kkv colocalise apically in tracheal cells

To find evidence of a functional interaction between Kkv and Exp/Reb, we analysed the subcellular localisation of these proteins. Exp immunostainings showed that the protein first accumulated in the cytoplasm of tracheal cells (in the temporal pattern described in Fig. 1A-C), but from stage 13–14 it also accumulated apically, lining the lumen, and later this apical accumulation was conspicuous (Fig. 5P). Reb protein was also enriched apically in the DT (Fig. 1E, 5Q) and in the rest of branches when misexpressed (Fig. 4B, C, G, H).

To analyse the accumulation of Kkv, we expressed a UASkkvGFP line in the trachea. We observed cytoplasmic accumulation of KkvGFP, but also a clear apical accumulation. KkvGFP colocalised with Exp and Reb in the apical region (Fig. 5P, Q).

### Together *reb* and *kkv* are sufficient to promote chitin deposition

As described above, the misexpression of *reb* brings about early and increased chitin accumulation in the trachea. In contrast, we found that *kkvGFP* overexpression does not induce, increase, or advance chitin deposition in the trachea or other tissues (S5A-C Fig.).

**Figure 6:**
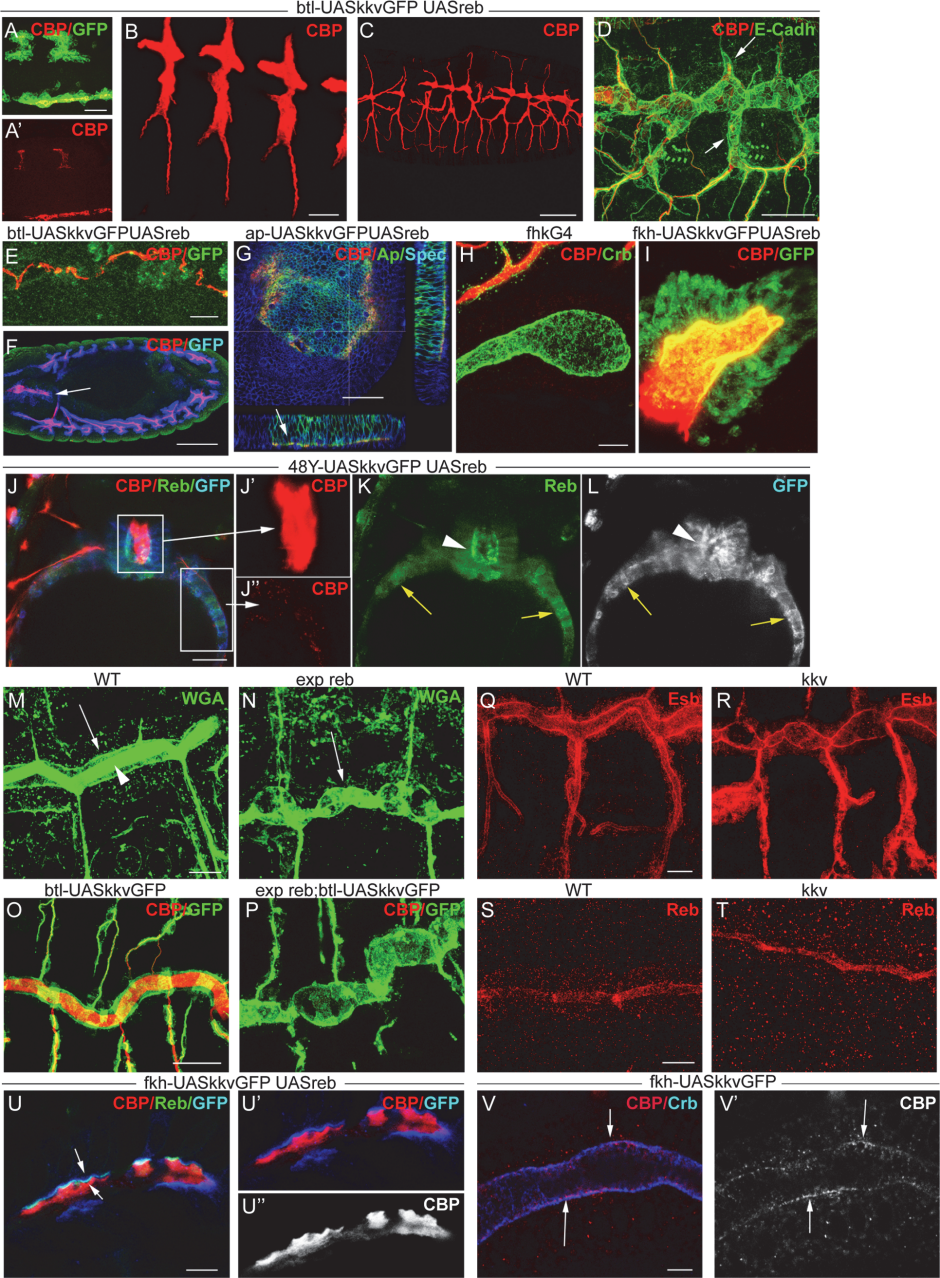
*reb* and *kkv* overexpression effects. All images are projections of confocal sections, except G, U and V, which are single sections. *reb* and *kkvGFP* overexpression in the *btl* pattern (in green in A) brings about early chitin deposition in the trachea (A’,B), which leads to a defective branching pattern (C) and defects of intercalation (arrows in D). In addition, it promotes chitin deposition in midline (A,E) and proventriculus (arrow in F). Misexpression of the two genes in imaginal discs (G) or salivary glands (H,I) also induces chitin deposition. *reb* and *kkvGFP* misexpression in the intestinal tract identified clear differences between the midgut (of endodermal origin) and the proventriculus (of ectodermal origin) (J). In the proventriculus, KkvGFP and Reb localised apically and chitin deposited in the lumen (J’,K,L). In the midgut, which is continuous with the proventriculus, Reb accumulation was cytoplasmic and KkvGFP localised in the entire cortical region (K,L). Chitin was not deposited extracellularly but few chitin particles we often detected (J’). Chitin precursors accumulate apically (arrow in M) and in the lumen (arrowhead in M). In *exp reb* mutants they accumulate apically (arrow in N). KkvGFP is found apically in control and *exp reb* mutants (O,P). In *kkv* mutants, Exp and Reb are expressed and accumulated apically (Q-T). Reb (U) colocalises with KkvGFP (U, U’) at the apical membrane when misexpressed in SGs, and chitin fibers (red in U,U’; white in U’’) are deposited extracellularly in the lumen. (V) When only *kkvGFP* is misexpressed in the SGs, no luminal chitin fibers are found but instead chitin particles (red in V, white in V’) are enriched in the apical and membrane region. Scale bars A,D,G,J,O 25 μm, B,E,H,Q 10 μm, C 50 μm, F 75 μm, M,U 7.5 μm, V 5 μm.

We simultaneously overexpressed the two genes in the trachea. Chitin accumulated even earlier (already at stage 11) and more strongly than when Reb was overexpressed alone (Fig. 6A, B). The increased chitin accumulation correlated perfectly with dramatic morphogenetic defects, as the tracheal branches became short, very straight, and generally unfused (Fig. 6C-D). Staining with E-Cadh revealed defects in tracheal cell shape organisation and cell intercalation (DB, LT and VB, which in the wild type form autocellular junctions [27], remained non-intercalated with intercellular junctions) (Fig. 6D). These results show that together these two genes have the capacity to trigger chitin deposition and that the early and excess deposition of chitin blocks tracheal morphogenesis.

More strikingly, we found that concomitant expression of *kkvGFP* and *reb* triggers chitin deposition in ectodermally-derived tissues that normally do not hold this polysaccharide. For instance, when using the *breathlessGal4* (*btlGal4*) driver, chitin deposited in tissues in which it does not normally accumulate, such as the midline and the proventriculus, sites in which the driver is expressed (Fig. 6E-F). Chitin also highly accumulated in salivary glands (SGs) when using a *forkheadGal4* (*fkhGal4*) line. SGs accumulating luminal chitin did not properly invaginate or undergo extension (Fig. 6H-I). Expression in the dorsal part of imaginal discs (*apterous* (*ap*) domain) promoted apical accumulation of chitin between the peripodial membrane and the epithelia (Fig. 6G), which led to strong morphogenetic defects in the adult wing and notum (S5D-E Fig.). Similar but milder effects were observed when expressing *kkvGFP* and *exp* (S3G-H Fig.).

These results show that the simultaneous activities of Kkv and Exp/Reb are sufficient to promote chitin deposition in the ectoderm, even in tissues in which chitin does not normally accumulate, thus blocking morphogenesis.

We evaluated the capacity of *kkv* and *reb* to trigger chitin synthesis when concomitantly misexpressed in mesodermal or endodermal tissues. We found no extracellular chitin deposited in those tissues (Fig. 6J-L). A detailed inspection indicated that, in contrast to what happens in ectodermal tissues, Reb did not localise apically in these non-ectodermal tissues and instead accumulated in the cytoplasm. KkvGFP was enriched in the entire cortical region. This result indicates that the apical accumulation of Reb requires factors present only in ectodermal tissues or the general ectodermal apicobasal polarity. It also indicates a clear correlation between the subcellular localisation of these proteins and chitin deposition.

We also evaluated the activity of *kkv* and *reb/exp* in cell culture in vitro assays (S6 Fig.). We transfected S2 cells and found that the presence of KkvGFP leads to the formation of small chitin-containing particles intracellularly. The pattern of these particles was not changed when the cells transfected with *kkvGFP* were also cotransfected with *exp, reb*, or *exp+reb*, and we never detected fibrilar chitin deposited extracellularly. Interestingly, we found that Kkv and Exp/Reb did not localise in the cell membrane region. The results in cell culture are in line with our in vivo experiments in non-ectodermal tissues, and further support a correlation between the subcellular localisation of these proteins and their activity in chitin deposition.

### Mechanism underlying Exp/Reb activity

We examined the participation of Exp/Reb in chitin deposition. For this purpose, we first analysed whether the sugar precursors (UDP-GlcNAc) were present in the *exp reb* mutants. The lectin wheat germ agglutinin (WGA) recognises the terminal GlcNAc residues of chitin [29]. In the wild-type trachea, WGA strongly accumulated in the lumen and apical surface (Fig. 6M). In mutants, WGA was largely absent from the lumen but still accumulated in the apical region (Fig. 6N). This observation strongly suggests that, as occurs in *kkv* mutants [4], *exp/reb* are not required for the synthesis or apical accumulation of GlcNAcs.

Exp/Reb and KkvGFP colocalisation results suggested an interdependent apical accumulation. However, KkvGFP still accumulated apically in the absence of *exp reb* (Fig. 6O-P), and Exp and Reb were also apical in *kkv* mutants (Fig. 6Q-T). These findings indicate that Kkv and Exp/Reb accumulate apically, lining the lumen in an independent manner.

The ectopic expression of *kkv* and *reb* in the SGs (which comprise large, single-layered epithelial cells that form a tube) gave some clues about the possible mechanism. As indicated, when we simultaneously expressed *kkvGFP* and *reb*, fibrilar chitin was deposited extracellularly in the lumen of the SGs, while Kkv and Reb colocalised in the apical membrane (Fig. 6U). In contrast, when we expressed only *kkvGFP* at high levels in the SGs, chitin was not deposited in the lumen. However, we observed the presence of small chitin particles (marked with CBP) highly enriched in the apical and/or membrane region (Fig. 6V, S5F-G). These chitin particles did not colocalise with cytoplasmic *kkvGFP* vesicle-like particles (S5G Fig.). Occasionally, we also detected the presence of chitin particles in the apical region of tracheal cells overexpressing *kkvGFP* but mutant for *exp/reb* (S5H Fig.). It has been proposed that chitin deposition requires first the formation of polymers, their translocation across the membrane to the extracellular space, and finally their assembly into microfibrils [17,18,30]. In light of this, a possible interpretation of our results is that, in the presence of Kkv, small chitin polymers are synthesized but cannot be extruded extracellularly and/or further organised into microfibrils without the activity of Exp/Reb.

## Discussion

Here we identified two MH2-containing genes, *exp* and *reb*, that play a key role in morphogenesis in *Drosophila*. Their absence gives rise to an abnormal and physiologically inactive tracheal system and defective epidermal cuticle formation. These defects are identical to those of mutants for the chitin synthase Kkv. Our analysis has shown that, like Kkv, Reb/Exp are absolute requirements for the specific step of chitin deposition, and that without Reb and Exp, Kkv cannot perform this activity. Furthermore, over- and mis-expression experiments showed that Exp/Reb are not only required but are also sufficient to bring about early and increased chitin deposition in the presence of Kkv. Strikingly, we also found that when *exp/reb* and *kkv* are simultaneously misexpressed in tissues in which chitin is not normally deposited, they trigger the accumulation of this polysaccharide. It was already known that chitin deposition is critical for growth and development [7,25]. Here we show that, in addition, ectopic, premature or excess deposition of chitin leads to dramatic defects in morphogenesis.

Identification of putative Exp orthologs in other arthropods and in the nematode *C. elegans* (which also synthesizes chitin, [31]), and the requirement of the respective gene for viability of *T. castaneum*, together suggest a conserved essential role of these proteins in chitin deposition in ecdysozoans. Interestingly, Exp and its orthologs seem to control production and orientation of chitin especially in ectodermal tissues. Indeed, *exp* and *reb* are not expressed in the endodermal midgut cells that nevertheless synthesize chitin as an important element of the protective peritrophic membrane. Consistently, misexpression of *kkv* and *reb* in midgut cells do not promote extracellular chitin deposition. This result indicates that midgut cells are incompetent to support Exp and Reb function.

Is there any explanation for the specific requirement of Exp and Reb in ectodermal tissues? We hypothesize that the function of Exp and orthologs correlates with the extracellular organisation of chitin. In insects, ectodermal chitin eventually interacts with specific proteins to form a three-dimensional matrix in the cuticle and the tracheal lumen [3]. By contrast, peritrophic chitin, produced independently of Exp and Reb, associates with another set of proteins rather forming a plain lattice [32]. Likewise, orthologs of Exp/Reb are not found in fungi like yeast and *Neurospora crassa* that form a simple chitin layer at the basal zone of their cell wall [33,34,35].

Together, these observations suggest that chitin synthesis has acquired different molecular requirements during evolution and that these proteins are a new invention to confer specific properties to particular type of chitin-based matrices. Given that chitin is present in all insects, its synthesis represents an excellent target for the control of insect pests [36]. Therefore, our findings may provide relevant information for the design of new drugs and insecticides, with no undesired effects on vertebrates.

### Exp and Reb are functionally interchangeable

Both Exp and Reb are required for chitin deposition. While no chitin is deposited in the trachea in the absence of both genes, the presence of one or the other can fully rescue this phenotype. Thus these two genes can perform the same function and they are interchangeable. The pattern of expression and functional requirements of each of these genes in normal conditions illustrate an elegant mechanism of activity. The phenotype of *exp* down-regulation (in all branches except the DT) is complementary to the pattern of *reb* (expressed only in the DT from stage 13). Our results show that Reb allows chitin deposition in the DT in the absence of Exp. We also reveal that the removal of *reb* causes only a delay in DT chitin deposition, due to the presence of Exp in the DT from stage 14 onwards. Thus *exp* and *reb* are redundant when expressed in the same tissue.

**Figure 7:**
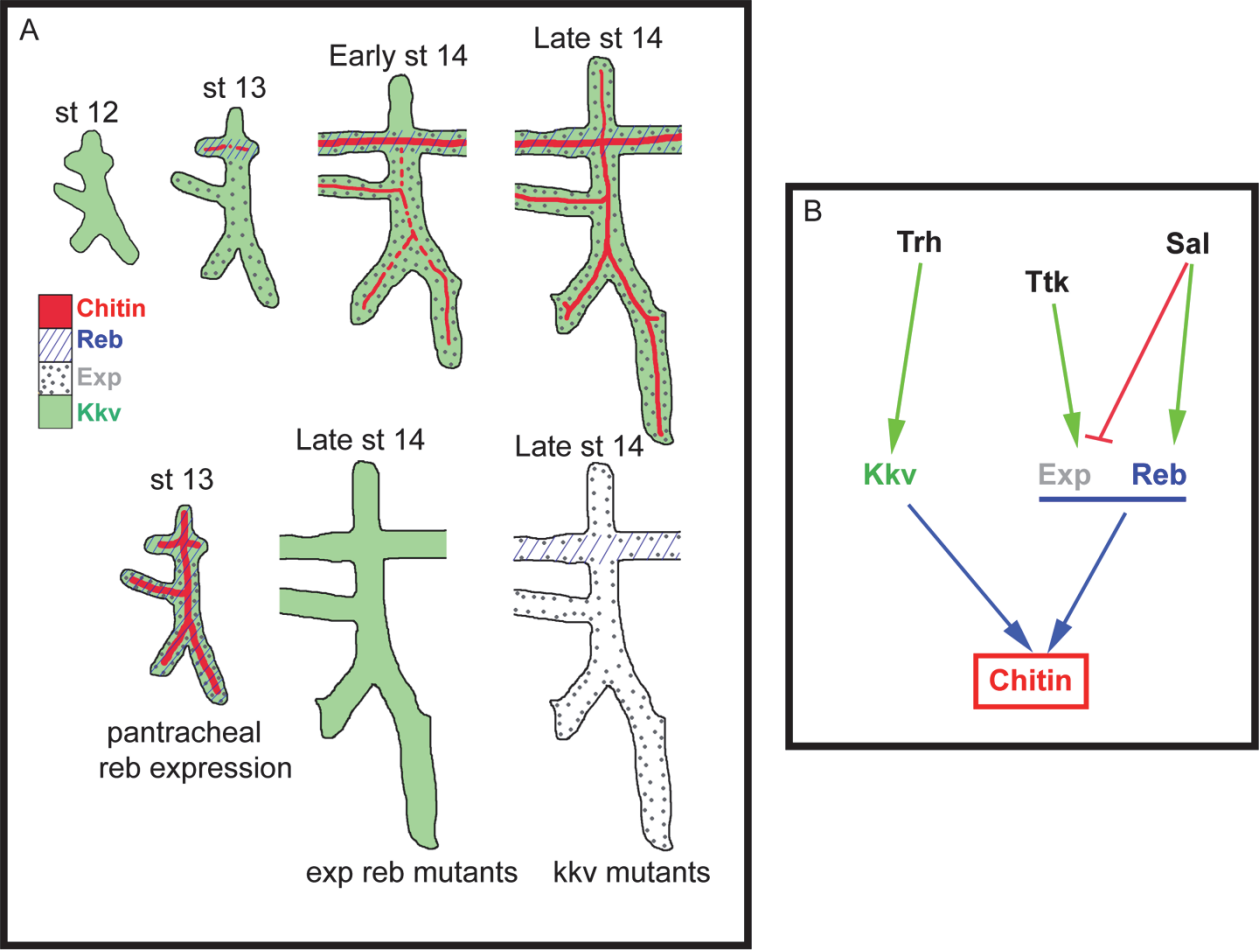
Model of luminal chitin deposition in the trachea. (A) Kkv is present from early stages in all tracheal cells. At stage 13 Reb appears in the DT and Exp in the ventral part of the placode. Chitin accumulation starts in the DT region, where Kkv and Reb are concomitantly expressed. At early stage 14, chitin starts being deposited in regions of concomitant expression of Kkv and Exp. Exp expression progressively expands to the dorsal part of the placode, correlating with the deposition of chitin there. When Reb is expressed earlier and in all tracheal cells, chitin precociously and strongly accumulates in all branches. When Exp and Reb are missing, the presence of Kkv cannot trigger chitin deposition, and vice versa. (B) Scheme of the transcriptional regulation of *kkv, exp*, and *reb* in the trachea that ensures the timely and spatially regulated accumulation of chitin.

What is the functional relevance of having two genes with interchangeable roles in chitin deposition and that show partially overlapping expression? An unequivocally answer is difficult, but several lines of evidence are worth considering. On the one hand, we found that *reb* expression is restricted to the DT during embryogenesis, suggesting that it is required only to ensure early and strong chitin deposition in this region. This is consistent with our results, showing that in the absence of *reb*, DT chitin deposition is delayed and decreased, while the rest of branches and the cuticle are not affected. On the other hand our findings suggest that Reb is more efficient than Exp in performing the same function. Indeed, strong over or misexpression of *reb* alone or in combination with *kkv* generated stronger effects than when strongly overexpressing *exp*. In addition, in normal conditions, chitin is first deposited in branches that express *reb* (i.e. the DT), in spite the fact that *exp* is also expressed at the same time in other branches (see Fig. 7A). In contrast to *reb, exp* is expressed in all tracheal cells and in the rest of chitin-synthesising tissues and is required for general chitin deposition. Hence, we hypothesise that Exp is more general but less efficient at promoting chitin deposition, while Reb is more restricted but more efficient. Various explanations could be given regarding the differences in efficiency, ranging from differences at the functional level to differences in the subcellular localisation (we note that Reb apical localisation is more conspicuous than that of Exp, both in normal and overexpression conditions). In summary, Reb appears to be required only when a rapid and strong accumulation of chitin is needed. The deposition of chitin first in the DT region may represent an advantage, particularly considering that the DT does not undergo cell intercalation [27], a process that is impaired when excess chitin accumulates. Thus, the earlier and stronger accumulation of chitin in the DT may be a safety mechanism which serves to prevent cell intercalation, thereby allowing normal morphogenesis.

### The role of Exp and Reb in chitin deposition

Chitin deposition depends on CHS enzymes present in all chitin-synthesising organisms. In spite of the relevance of CHSs, their exact mechanism of activity remains obscure [17,18].

Kkv encodes the epidermal and tracheal CHS in *Drosophila* [5,15,25]. Kkv is transcriptionally expressed in tissues that normally deposit chitin, i.e. the tracheal system and the epidermis. In the trachea, Kkv is present in all tracheal cells from late stage 11-early stage 12 until late embryonic stages [5] and BDGP in situ homepage (http://insitu.fruitfly.org). *kkv* is an early target of Trachealess (Trh) activity [37]. In spite of the general and early *kkv* tracheal expression, the deposition of chitin does not start until stage 13, when it occurs in a spatio-temporal restricted manner (Fig. 7B, S2A-H). This observation indicates that Kkv activity is subjected to post-transcriptional regulation.

We conclude that the regulated spatiotemporal expression of *exp* and *reb* perfectly accounts for this post-transcriptional regulation of Kkv activity. This conclusion is based on two findings. First, Sal-dependent *exp/reb* expression in the trachea is consistent with the temporal pattern of chitin deposition by Kkv, whose expression is independent of Sal; and second, Kkv is only sufficient to promote chitin deposition (both in chitin-synthesising and chitinless tissues) in the presence of Exp/Reb. Therefore, chitin is deposited only in those cells that concomitantly express *kkv* and *exp/reb* (Fig. 7A-B).

The unexpected incapacity of Kkv to trigger chitin deposition on its own may reflect the need of a mechanism that ensures chitin accumulation only when and where it is required. It has been proposed that chitin deposition by the midgut-specific chitin synthase-2 (CHS2) in the midgut of the tobacco hornworm *Manudica sexta* depends on a chymotrypsin-like protease. It has been hypothesized that the proteolytic regulation of CHS-2 activity, which promotes the synthesis of chitin needed to protect the midgut epithelium against damage, relies on the nutritional state [38,39]. As expected, the situation is different in the epidermis and the trachea. If Kkv were sufficient to promote chitin deposition, any temporal or spatial misregulation of its expression would lead to ectopic chitin deposition, which we show causes serious morphogenetic defects. Interestingly, we found that *kkv* and *reb/exp* expression patterns are regulated in distinct manners (at least in the trachea) (Fig. 7B). This differential regulation may represent a way to restrict chitin accumulation in a coordinated manner, providing a complex control mechanism for final organ maturation.

What is the molecular mechanism of chitin deposition? Kkv and Exp/Reb accumulate in the apical membrane in tissues that normally deposit chitin, which have an ectodermal origin. Our experiments indicate that when Kkv and Reb are present, but are not apical (e.g. when misexpressed in the endoderm, or in S2 cells), no extracellular chitin deposition is detected. These observations show that only ectodermally-derived tissues contain the molecular machinery or the adequate apicobasal polarity to properly localise or maintain these proteins apically localised. In addition, the correlation between the subcellular localisation of these proteins and extracellular chitin deposition strongly suggests that the function of Kkv and Exp/Reb is required in the apical membrane. Thus, the subcellular localisation of these proteins could represent an extra level in the regulation of chitin deposition. In line with this hypothesis, we speculate that the inability of Reb and Kkv to accumulate apically in the membrane prevents extracellular chitin deposition in non-ectodermal tissues.

What is the role of Exp/Reb in chitin deposition? Chitin formation has been proposed to follow three steps. In the first step, the catalytic domain of the CHS that faces the cytoplasm forms polymers. In the second, the nascent polymers are translocated through the membrane to the extracellular space. Finally, in the third step the polymers spontaneously assemble to form crystalline microfibrils [17,18,30]. When we strongly expressed *kkv* in the absence of *exp/reb*, we observed an apical/membrane enrichment of small chitin-containing particles that appear to be unable to be deposited extracellularly or to form microfibrils. When we added *exp/reb* to this background, chitin was deposited extracellularly in a fibrillar organisation. These results suggest that Exp/Reb may participate in the steps of polymer translocation across the membrane and/or in microfibril formation. Exp/Reb do not hold canonical transmembrane domains, suggesting that they could interact or recruit, through their MH2 domain, other proteins directly involved in the translocation. It has been proposed that the carboxy-terminal region of CHS is involved in membrane translocation of polymers [30], thus raising the possibility that Exp/Reb interact with this domain. Alternatively, Exp/Reb may be required to directly or indirectly (by promoting the formation of a complex) activate CHS posttranscriptionally. Several postranscriptional modifications have been proposed for CHS, such as oligomerisation, phosphorylation, proteolytic cleavage (of a soluble factor that activates chitin synthesis), and the release of the nascent polymers to form microfibrils [17,18,38,40]. Exp/Reb may participate in these modifications.

In summary, altogether our results indicate that chitin deposition needs to be highly regulated in time and space, and that the finely tuned regulation of chitin deposition relies on a spatiotemporal control of the activity of Exp/Reb controlling Kkv-dependent chitin deposition. Thus, here we have identified the genetic programme required for timely and

## Materials and Methods

### *Drosophila* strains and genetics

The fly strains used are described in FlyBase: Df(2R)BSC329, Df(2R)ED2247, Df(2R)BSC879, Df(2L)32FP-5 (removes *sal-m* and *sal-r*), *kkv^IB22^*, and *kkv^63-20^*. The transgenes used were: P{TRiP.HMS01445}attP2, P{TRiP.HMS01444}attP2, P{KK111583}VIE-260B, P{GD7952}v17126, P(GSV6)GS15050, P(Mae-UAS.6.11)LA00773, and UAS-ANFGFP.

For overexpression experiments, we used the following Gal4 drivers: *btlGal4* (in all tracheal cells), *fkhGal4* (in salivary glands), *69B* (generally epidermal expression), *tubGal4* (general expression), *apTomato-Gal4; Gal80ts* (in the dorsal part of the imaginal wing disc), *48YGal4* (in the intestinal tract) and *twiGal4* (in the mesoderm). To maximise the expression of the transgenes crosses were kept at 29°C.

To visualise the “tracheal pattern”, the embryos carrying *btlGal4 UAS-srcGFP* (cell membrane staining) were stained for GFP. The *bltGal4* in this combination also drives other UAS transgenes

### Generation of UAS transgenes

Transgenic flies carrying *UAS-CG13188, UAS-CG13188-HA, UAS-CG13183*, and *UAS-kkvGFP* were generated (see S1 Text for details)

### Cuticle preparation

Fully developed embryos were dechorionated in bleach, devitellinized by shaking in 100% methanol, and incubated over night at 65°C in Hoyer’s medium mixed with lactic acid (1:1). Embryos were analysed by light microscopy using a Nikon Eclipse 80i microscope.

### Gas filling analysis

To evaluate gas filling in the tracheae, we followed the procedure described in [41]

### EM analysis

For ultrastructural analyses by transmission electron microscopy (TEM), wild-type and *exp reb* mutant embryos were immobilised by high-pressure freezing, fixed by freeze substitution, embedded in Epon, and sectioned as described previously [26]. Images were taken on a CM10 electron microscope.

### Immunohistochemistry and *in situ* hybridization

We followed standard protocols for immunostainings and *in situ* hybridisations. Embryos were staged as described [42]. Imaginal discs were obtained by dissecting third instar larvae.

The following primary antibodies and dilutions were used: mouse anti-2A12 (recognises Gasp, 1:10), rat anti-DEcad (1:100), and mouse anti-Crb (1:20) from Developmental Studies Hybridoma Bank, DSHB; rbb anti-Verm (1:300) from S. Luschnig; goat anti-GFP (1:600) Molecular Probes and Roche; ck anti-ßGal (1:500) abCAM; GP anti-Uif (1:400) from R. Ward; and rbb anti-Pio (1:100) from M. Affolter. CBP (chitin-binding probe) conjugated with Cy3, Cy2 and Cy5 was used at 1:300 (generated by N. Martin). WGA conjugated with Alexa-555, -488, and -647 was used at 1:300 (Molecular Probes). Cy3-, Cy2- and Cy5-conjugated secondary antibodies (Jackson ImmunoResearch) were used at 1:300.

A *reb* riboprobe was generated using the following primers:

Forward: 5′- AACTGTGCCTCGGCGCTAGTC
Reverse: 5′- AGCAGTCGAAACACGCAGCTT

Confocal images were acquired with a Leica TCS-SPE system. Images were post-processed with ImageJ and Adobe Photoshop and assembled using Adobe Illustrator.

### Generation of CG13188 and CG13183 antibodies

Polyclonal antibodies against CG13188 and CG13183 were generated (see S1 Text for details). Purified recombinant proteins were used as antigens to immunise rats and rabbits following standard protocols.

### S2 cells transfection experiments

*Drosophila* S2 cells were transiently transfected using Cellfectin II Reagent (Invitrogen). Cells were cultured in Schneider’s Insect Medium (Sigma) enriched with 10% of FBS (Fetal Bovine Serum, Gibco) and were used for immunostaining assays after 3 days of expression. Reb and Exp cDNAs were obtained by PCR from RE66796 and RE28239 clones, respectively (DGRC, Bloomington, IN). Kkv (with or without GFP) cDNA was obtained by PCR from UAS-GFPkkv flies (B. Moussian). In experiments without KkvGFP, DNA constructs were co-transfected with pAc5.1-GFP (a gift from J. Bernués) to visualise expressing cells. The fragments were cloned into pMT or pAC5.1/V5-His A (Invitrogen) expression vectors.

### Quantitative PCR

Total RNA from control and exp RNAi embryos at stages 11 to 16 was used to synthesise complementary cDNA by random hexamer priming (RevertAid H Minus First Strand cDNA Synthesis FERMENTAS Kit). A LightCycler 480 Real-Time PCR System and the SYBR Green PCR Master Mix (Roche) were used to amplify cDNAs. CG13167, a mitochondrial ATPase with stable expression, was used to normalise relative quantities. Samples were analysed using the LightCycler 480 Real-Time PCR System software (Roche) (see S1 Text for details).

## Supporting Information

S1 Fig Analysis of *exp* downregulation. (A) *exp* RNA from control (btlsrcGFP) and *exp* downregulation conditions (btlsrcGFP-UASexpRNAi) overexpressed at 29° was quantified by qRT-PCR. Gene expression levels were normalised using the endogenous control mitochondrial ATPse CG13167 that showed stable expression in all conditions. Note the strong reduction of exp levels. Error bars indicate SD. (B-K) Projections of confocal sections of embryos at stage 15 or 16. L,M dark field and N,O bright field images. The tracheal down-regulation of *exp* allows a normal branching pattern (B,C), normal cell organisation (visualised by the junctional pattern, D,E) and normal cell specificaction (as DSRF-expressing terminal cells are normally formed F,G). In contrast, chitin is not deposited in dorsal and ventral branches (F,G) and chitin associated proteins like Verm are also absent from the lumen in dorsal and ventral branches (arrow in I), but present in the DT (arrowhead in I). Stainings with apical markers (like Uif, J,K) show the formation of apical expansions preferentially in the LT region (arrow in K). The embryonic cuticle is more inflated (compare M to L) and there are defects in the mouth region (N,O). Scale bars A 25 μm, J 10 μm.
(TIF)

S2 Fig Temporal pattern of tracheal chitin deposition. All panels show projections of confocal sections except J which shows a bright field image. Note the temporal pattern of chitin deposition (red in A-H, white in A’-H’) from stage 12 (A,A’) to late stage 14-early 15 (H,H’). The tracheal branches are labelled in green (A-H). Chitin appears first in the DT at st 13, stronger in the fusion region (arrow in B’). Subsequently it starts to accumulate in the VB (arrows in C’,D’) by early st 14. During st 14 it is deposited in the LT and TC (arrows in D’,E’), and soon after in the DB (arrows in F’,G’). Note the strong accumulation of chitin in the DT region, very conspicuous at late 14-early 15 (G’,H’). When *reb* is removed with a deficiency combination, the down-regulation of *exp* leads to the absence of chitin in all branches (I’), although the branches normally form (I). The chitin-defective trachea does not fill with air (J). Scale bars A 10 μm, I 50 μm.
(TIF)

S3 Fig Analysis of *exp* overexpression. All panels show projections of confocal sections of embryos at st 13 (A,C) or 15 (B,D,E,F, G,H). The overexpression of *exp* leads to a higher accumulation of Exp protein (A,B) and precocious chitin accumulation in the trachea (arrows in C). The tracheal defects of the overexpression of *reb* (identified by lacZ expression in the cross in E) are not rescued when adding UASexp (F). When *exp* and *kkv* are missexpressed in the SGs they promote luminal chitin deposition (arrow in H). Scale bars A 25 μm, B 50 μm, G 10 μm.
(TIF)

S4 Fig Comparison of *exp reb* and *kkv* phenotypes. (A-O, S,T) Projections of confocal sections of embryos at stage 15–16. (P-R) Bright field images. Note the similar tracheal defects of *kkv* and *exp reb* mutants. Apical markers (A-C) and pio (M-O) are acummulated in control and mutant conditions. In contrast, markers for proteins that normally accumulate in the luminal chitinous filament, like Verm (G-I) or Gasp (D-F) are not properly found in the lumen (arrows in H,I). ANFGFP allows visualisation of secretion, and accumulates in the lumen at late stages (arrow in J). In mutants, the cytoplasmic pattern of ANFGFP is normal but it is not secreted into the lumen (arrows in K,L). The head region display clear cuticle defects (P-R). The tracheal expression of *kkvGFP* rescues the defects of *kkv* mutants and luminal chitin deposition (S). However, it does not rescue chitin deposition (red in T, white in T’) defects produced by *exp* downregulation (note that DBs do not accumulate chitin, arrows in T,T’). Scale bar A,S 25 μm, T 10 μm.
(TIF)

S5 Fig *reb* and *kkvGFP* overexpression. (A-C, F-H) Projections of confocal sections showing the tracheal system (A,B,H) or single sections of SG (C,F,G). (D,E) Bright field images. The sole overexpression of *kkvGFP* does not produce detectable defects. Chitin is deposited in the normal pattern (first in the DT at st 13, arrow in A) and the tracheal pattern is not affected (B). Nor it is able to produce chitin (C’) when missexpressed at low levels in the SG (C). However, when *kkvGFP* is strongly missexpressed in the SG, chitin particles (arrows in F’,G’) are detectable in the apical and membrane region (F,G). The chitin particles do not colocalise with kkvGFP positive vesicles (encircled in G,G’). Chitin particles (arrow in H’) are also occasionally detected when overexpressing *kkvGFP* in the trachea of *exp reb* mutants. When *kkvGFP* and *reb* are missexpressed together they produce strong defects. Note the defects in the adult wing (D) and notum (defective bristles in E) when missexpressed in the dorsal (apterous domain) part of the wing imaginal disc. Scale bars A 25 μm, B 50 μm, C,F,H 10 μm
(TIF)

S6 Fig S2 cells transfection experiments. Confocal projections of S2 cells expressing the indicated constructs and stained for the indicated antibodies. In experiments without DNA transfection (A) or transfection with Reb or Exp (together with actinGFP to visualised transfected cells, B,C), no chitin is detected (A’-C’). Note that Exp and Reb do not localise at the cell membrane (insets in B,C). In cells transfected with KkvGFP (D-G), the protein is found in the cytoplasm, often enriched in intracellular vesicles. The presence of KkvGFP leads to the formation of chitin-containing particles in a perinuclear region, often colocalising with KkvGFP vesicles (D’-G’). The pattern of chitin particles do not change when the cells are co-transfected with Reb (E), Exp (F) or both Exp and Reb (G), indicating that of Exp/Reb in S2 cells do not trigger extracellular deposition of fibrilar chitin. Scale bar 10 μm.
(TIF)

S1 Text Supplemental materials and methods and supplemental information (alignment of homologous sequences from insects and non-insects). (DOC)
