# Correction: Deciphering the Genetic Programme Triggering Timely and Spatially-Regulated Chitin Deposition

**DOI:** 10.1371/journal.pgen.1005054

**Published:** 2015-03-17

**Authors:** The PLOS Genetics Staff

There are errors in the author affiliations. The affiliations should appear as shown here:

Bernard Moussian^1☯¤a^, Annalisa Letizia^2☯^, Guillermo Martínez-Corrales^2☯^, Bárbara Rotstein^2¤b^, Andreu Casali^3^ and Marta Llimargas^2*^

1. Animal Genetics, Interfaculty Institute for Cell Biology, University of Tuebingen, Tuebingen, Germany, 2. Institut de Biologia Molecular de Barcelona, CSIC. Barcelona, Spain, 3. Institute for Research in Biomedicine (IRB Barcelona), Barcelona, Spain

¤a. Present address: Institut de Biologie Valrose, UNS Université Nice Sophia Antipolis, Nice, France

¤b. Present address: Fachbereich Biologie/Chemie, University of Osnabrück, Osnabrück, Germany

☯ These authors contributed equally to this work.

* mlcbmc@ibmb.csic.es

The acknowledgments are incorrect. The acknowledgments should appear as shown here:
