# Supplementary figures and images for: Deciphering the Genetic Programme Triggering Timely and Spatially-Regulated Chitin Deposition
Source: PLoS Genet. 2015 Jan 24;11(1):e1004939. doi: 10.1371/journal.pgen.1004939 (PMC4305360; doi:10.1371/journal.pgen.1004939)

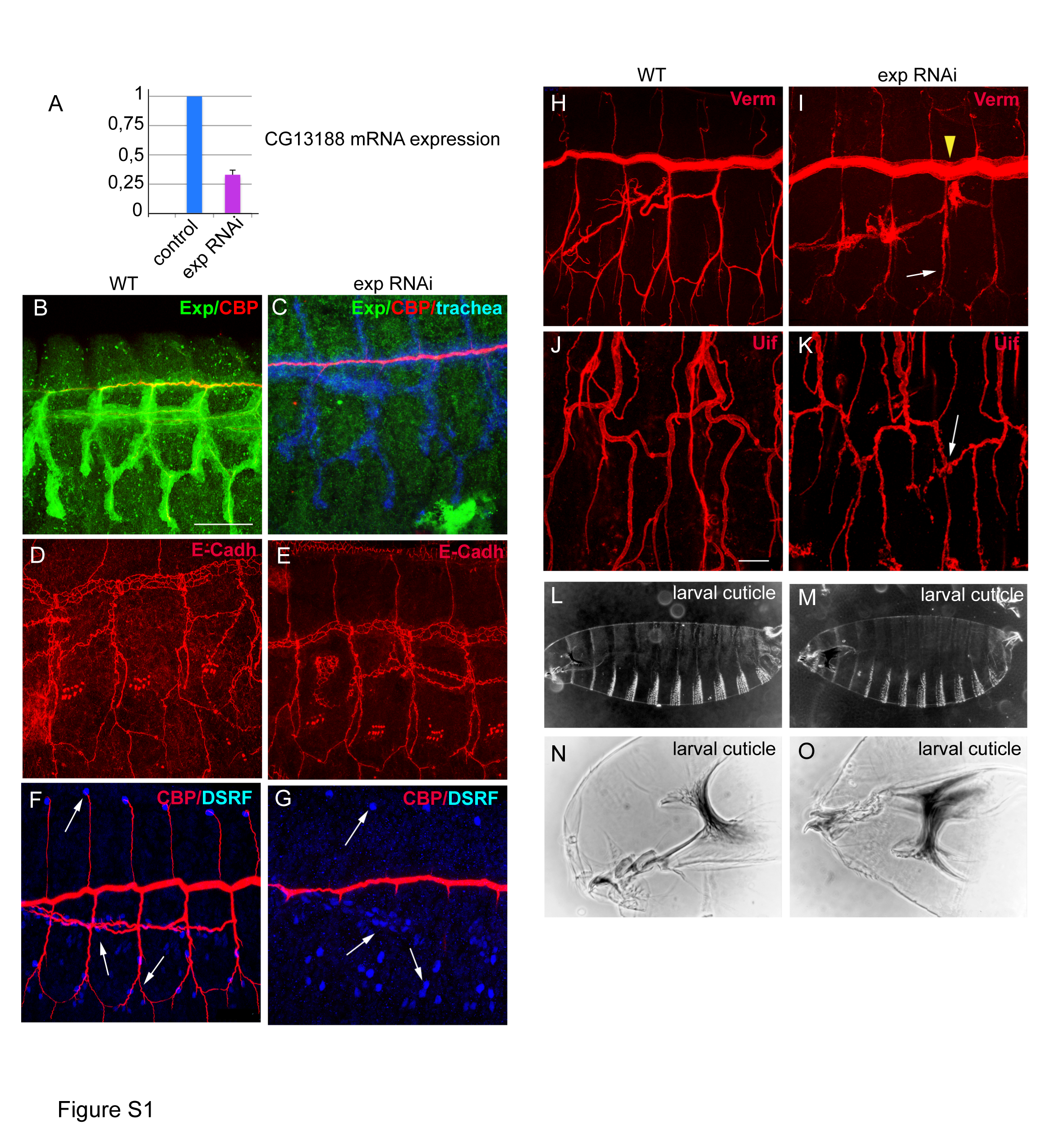

Supplement: S1 Fig — (A) exp RNA from control (btlsrcGFP) and exp downregulation conditions (btlsrcGFP-UASexpRNAi) overexpressed at 29° was quantified by qRT-PCR. Gene expression levels were normalised using the endogenous control mitochondrial ATPse CG13167 that showed stable expression in all conditions. Note the strong reduction of exp levels. Error bars indicate SD. (B-K) Projections of confocal sections of embryos at stage 15 or 16. L,M dark field and N,O bright field images. The tracheal down-regulation of exp allows a normal branching pattern (B,C), normal cell organisation (visualised by the junctional pattern, D,E) and normal cell specificaction (as DSRF-expressing terminal cells are normally formed F,G). In contrast, chitin is not deposited in dorsal and ventral branches (F,G) and chitin associated proteins like Verm are also absent from the lumen in dorsal and ventral branches (arrow in I), but present in the DT (arrowhead in I). Stainings with apical markers (like Uif, J,K) show the formation of apical expansions preferentially in the LT region (arrow in K). The embryonic cuticle is more inflated (compare M to L) and there are defects in the mouth region (N,O). Scale bars A 25 μm, J 10 μm. (TIF) [file pgen.1004939.s001.tif]

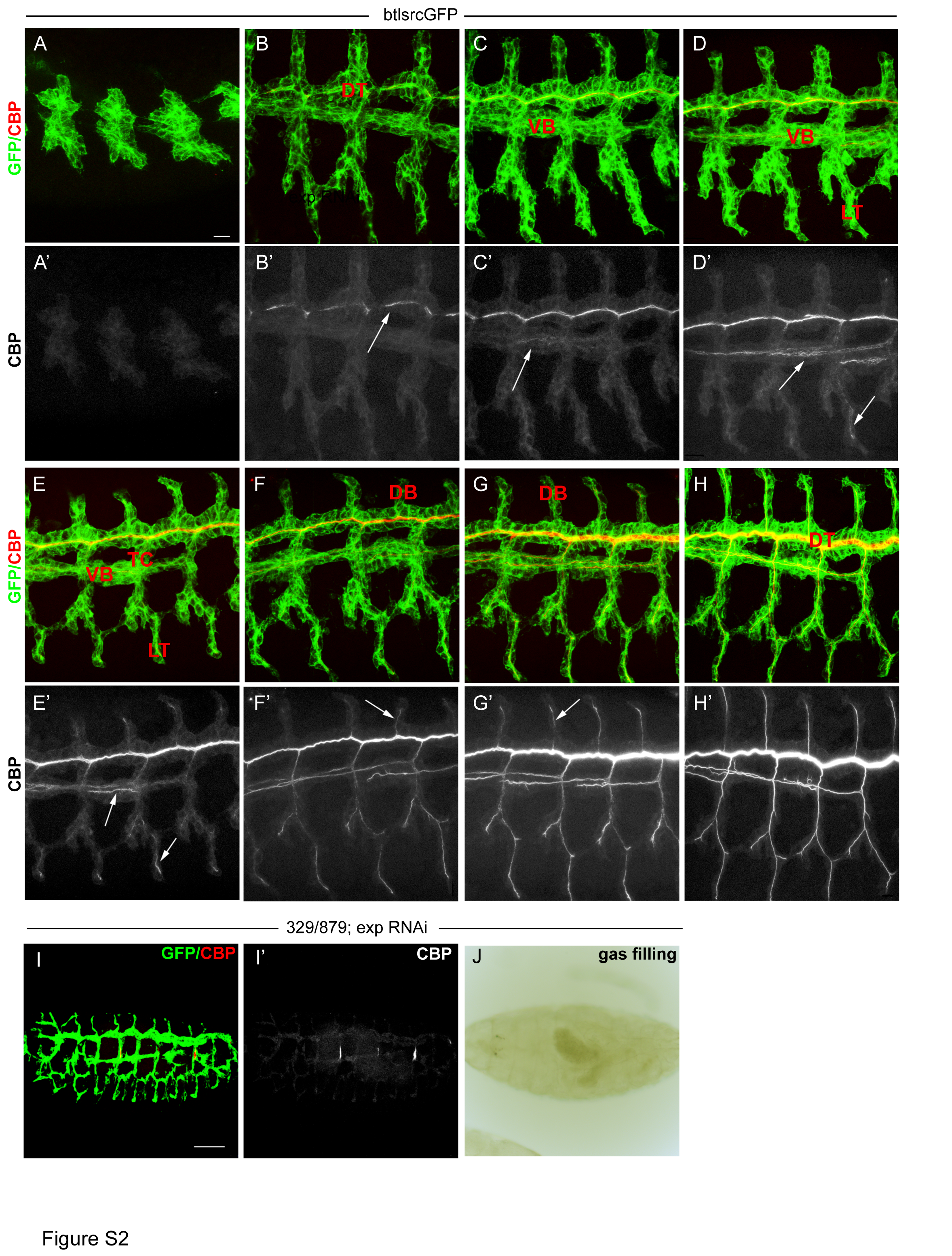

Supplement: S2 Fig — All panels show projections of confocal sections except J which shows a bright field image. Note the temporal pattern of chitin deposition (red in A-H, white in A’-H’) from stage 12 (A,A’) to late stage 14-early 15 (H,H’). The tracheal branches are labelled in green (A-H). Chitin appears first in the DT at st 13, stronger in the fusion region (arrow in B’). Subsequently it starts to accumulate in the VB (arrows in C’,D’) by early st 14. During st 14 it is deposited in the LT and TC (arrows in D’,E’), and soon after in the DB (arrows in F’,G’). Note the strong accumulation of chitin in the DT region, very conspicuous at late 14-early 15 (G’,H’). When reb is removed with a deficiency combination, the down-regulation of exp leads to the absence of chitin in all branches (I’), although the branches normally form (I). The chitin-defective trachea does not fill with air (J). Scale bars A 10 μm, I 50 μm. (TIF) [file pgen.1004939.s002.tif]

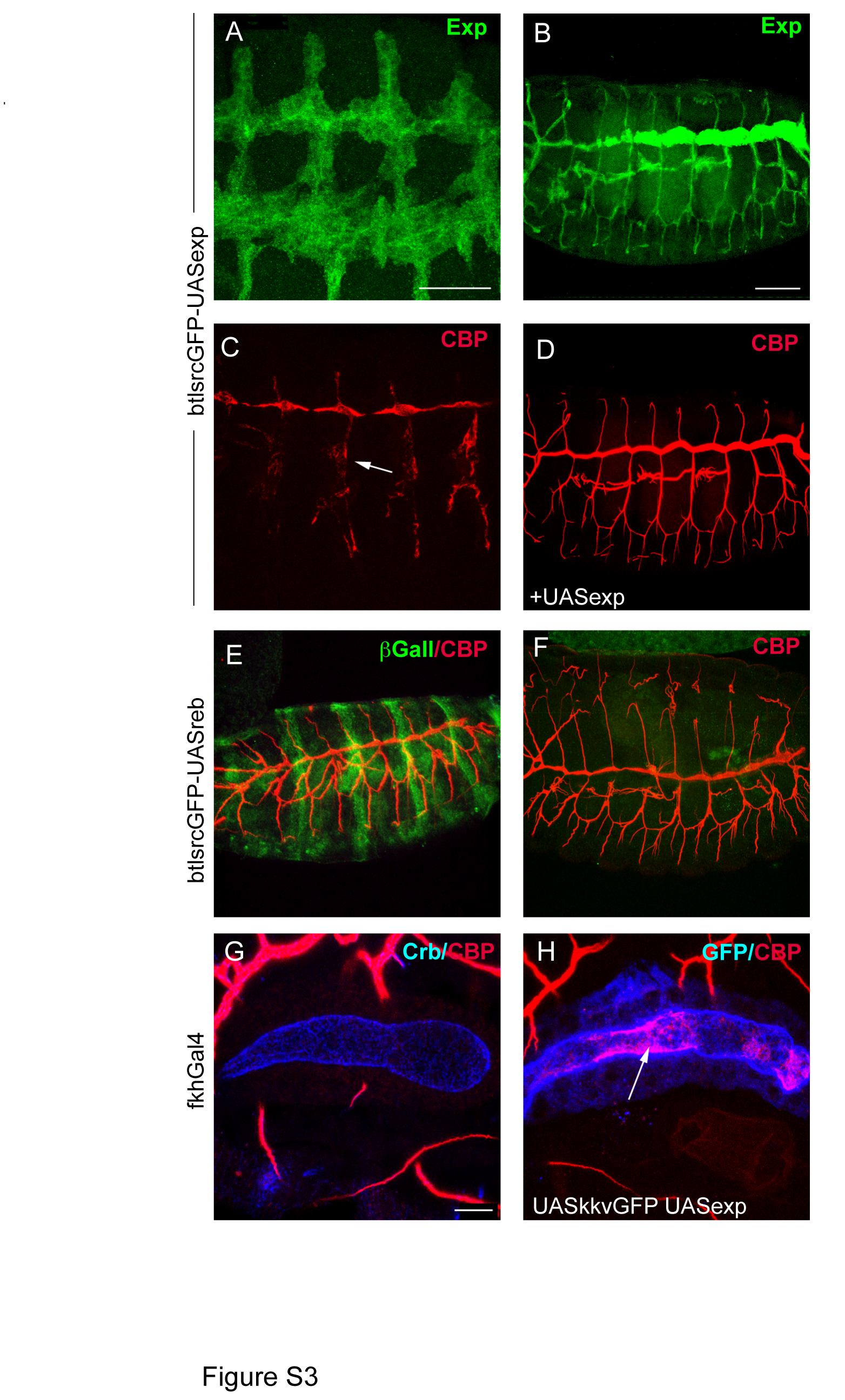

Supplement: S3 Fig — All panels show projections of confocal sections of embryos at st 13 (A,C) or 15 (B,D,E,F, G,H). The overexpression of exp leads to a higher accumulation of Exp protein (A,B) and precocious chitin accumulation in the trachea (arrows in C). The tracheal defects of the overexpression of reb (identified by lacZ expression in the cross in E) are not rescued when adding UASexp (F). When exp and kkv are missexpressed in the SGs they promote luminal chitin deposition (arrow in H). Scale bars A 25 μm, B 50 μm, G 10 μm. (TIF) [file pgen.1004939.s003.tif]

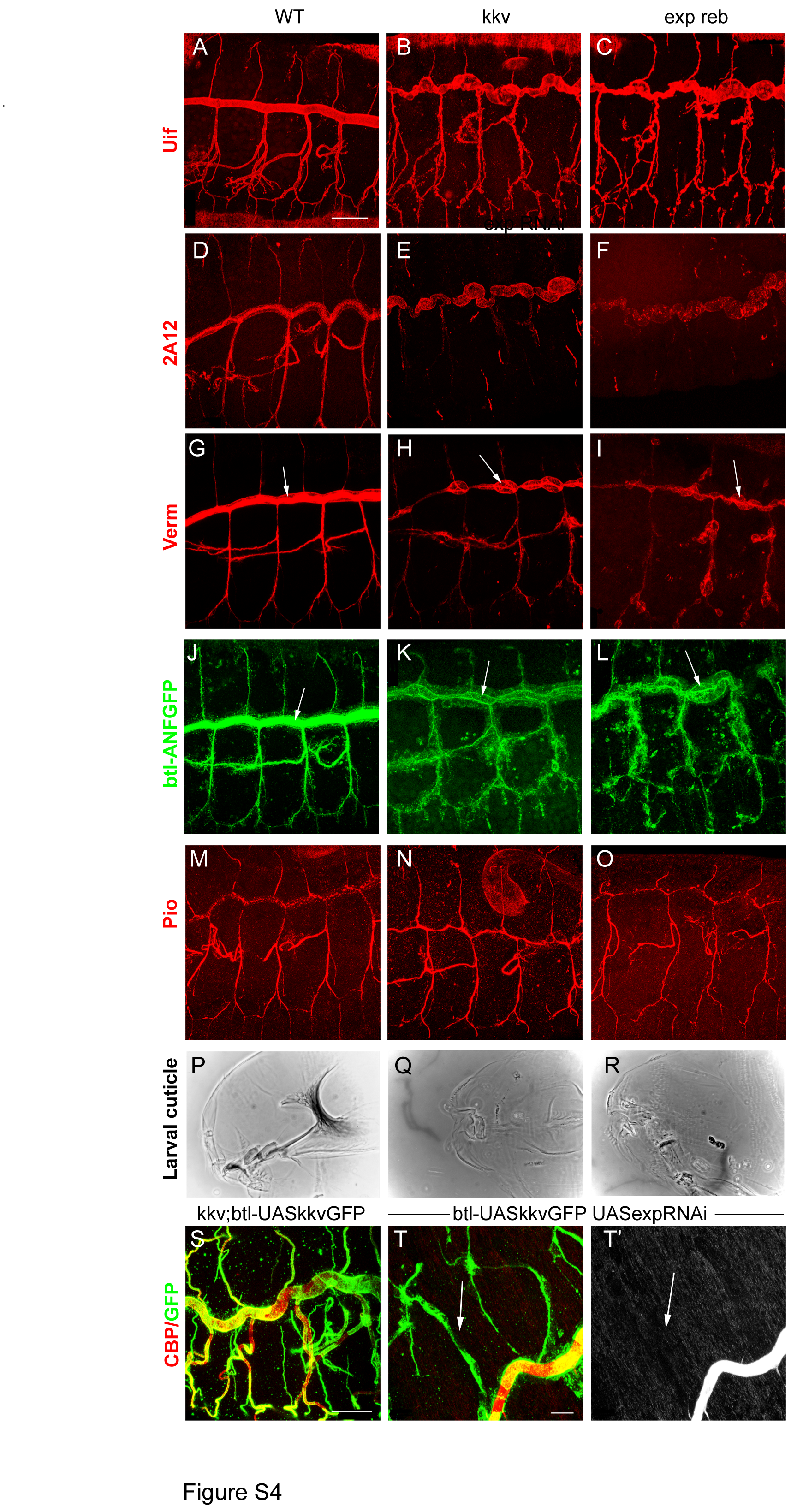

Supplement: S4 Fig — (A-O, S,T) Projections of confocal sections of embryos at stage 15–16. (P-R) Bright field images. Note the similar tracheal defects of kkv and exp reb mutants. Apical markers (A-C) and pio (M-O) are acummulated in control and mutant conditions. In contrast, markers for proteins that normally accumulate in the luminal chitinous filament, like Verm (G-I) or Gasp (D-F) are not properly found in the lumen (arrows in H,I). ANFGFP allows visualisation of secretion, and accumulates in the lumen at late stages (arrow in J). In mutants, the cytoplasmic pattern of ANFGFP is normal but it is not secreted into the lumen (arrows in K,L). The head region display clear cuticle defects (P-R). The tracheal expression of kkvGFP rescues the defects of kkv mutants and luminal chitin deposition (S). However, it does not rescue chitin deposition (red in T, white in T’) defects produced by exp downregulation (note that DBs do not accumulate chitin, arrows in T,T’). Scale bar A,S 25 μm, T 10 μm. (TIF) [file pgen.1004939.s004.tif]

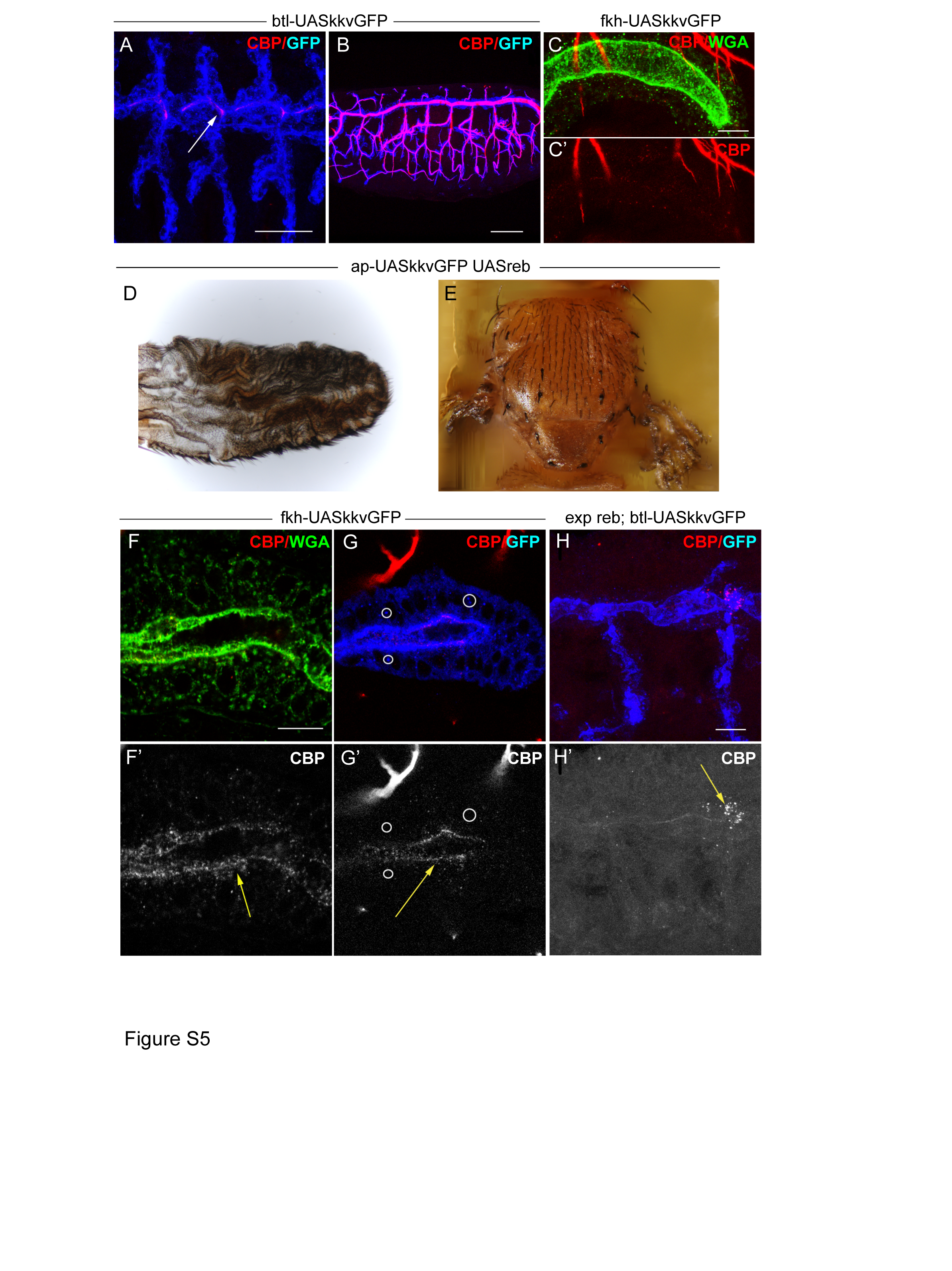

Supplement: S5 Fig — (A-C, F-H) Projections of confocal sections showing the tracheal system (A,B,H) or single sections of SG (C,F,G). (D,E) Bright field images. The sole overexpression of kkvGFP does not produce detectable defects. Chitin is deposited in the normal pattern (first in the DT at st 13, arrow in A) and the tracheal pattern is not affected (B). Nor it is able to produce chitin (C’) when missexpressed at low levels in the SG (C). However, when kkvGFP is strongly missexpressed in the SG, chitin particles (arrows in F’,G’) are detectable in the apical and membrane region (F,G). The chitin particles do not colocalise with kkvGFP positive vesicles (encircled in G,G’). Chitin particles (arrow in H’) are also occasionally detected when overexpressing kkvGFP in the trachea of exp reb mutants. When kkvGFP and reb are missexpressed together they produce strong defects. Note the defects in the adult wing (D) and notum (defective bristles in E) when missexpressed in the dorsal (apterous domain) part of the wing imaginal disc. Scale bars A 25 μm, B 50 μm, C,F,H 10 μm (TIF) [file pgen.1004939.s005.tif]

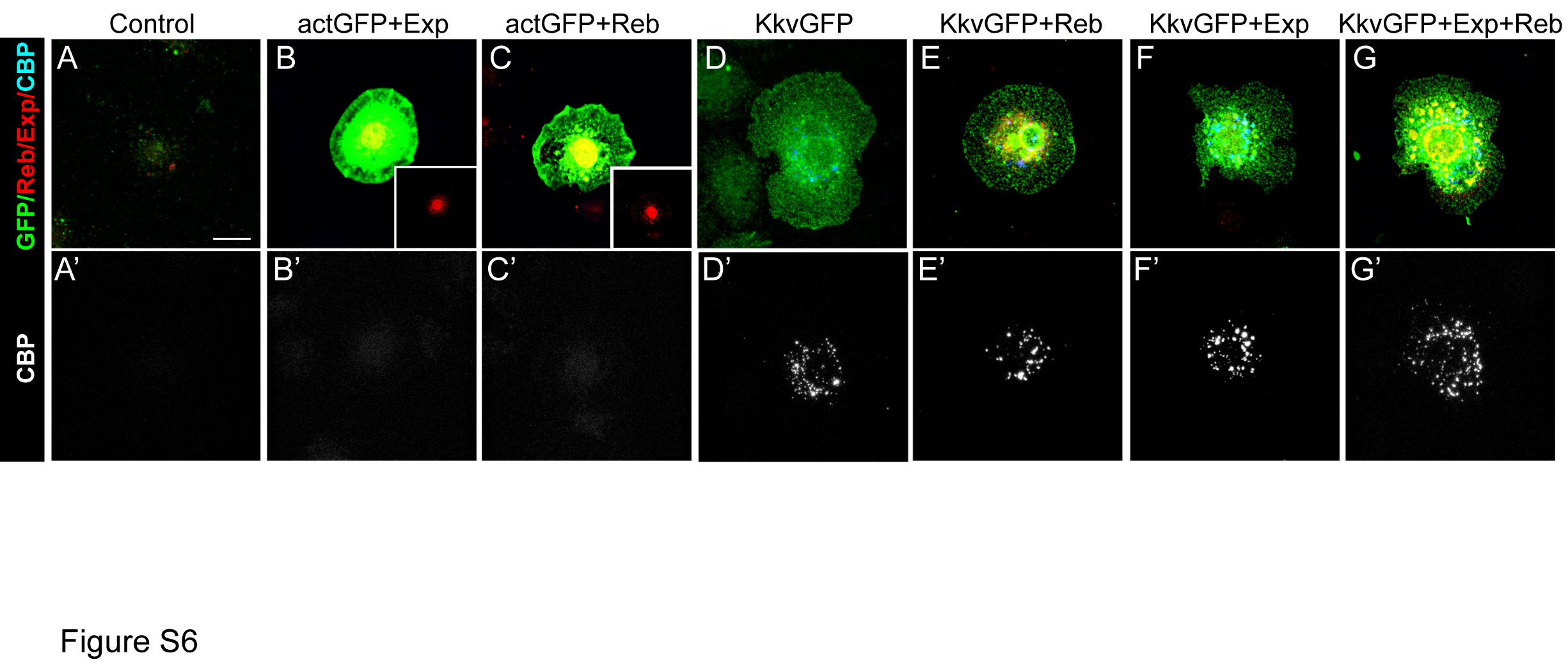

Supplement: S6 Fig — Confocal projections of S2 cells expressing the indicated constructs and stained for the indicated antibodies. In experiments without DNA transfection (A) or transfection with Reb or Exp (together with actinGFP to visualised transfected cells, B,C), no chitin is detected (A’-C’). Note that Exp and Reb do not localise at the cell membrane (insets in B,C). In cells transfected with KkvGFP (D-G), the protein is found in the cytoplasm, often enriched in intracellular vesicles. The presence of KkvGFP leads to the formation of chitin-containing particles in a perinuclear region, often colocalising with KkvGFP vesicles (D’-G’). The pattern of chitin particles do not change when the cells are co-transfected with Reb (E), Exp (F) or both Exp and Reb (G), indicating that of Exp/Reb in S2 cells do not trigger extracellular deposition of fibrilar chitin. Scale bar 10 μm. (TIF) [file pgen.1004939.s006.tif]
