# Supplementary material for: Deciphering the Genetic Programme Triggering Timely and Spatially-Regulated Chitin Deposition
Source: PLoS Genet. 2015 Jan 24;11(1):e1004939. doi: 10.1371/journal.pgen.1004939 (PMC4305360; doi:10.1371/journal.pgen.1004939)
Supplement: S1 Text — (DOC) [file pgen.1004939.s007.doc]

**SUPPLEMENTAL MATERIALS AND METHODS**

**Generation of UAS transgenes**

To generate UAS CG13188 lines we used the cDNA clone RE28239, that includes all the CG13188-RB protein. A PCR using the following primers was used:

Forward 5'- GGTACCCAAGCTTCGATGGTGTCGCGTCGAAAAATTCTATCACG -3'

Reverse 5'- TCTAGATCAGTCCCATTCCCCGATGAACAT- 3'

To generate UASCG13188-HA the primers used were:

Forward 5'- GGTACCCAAGCTTCGATGGTGTCGCGTCGAAAAATTCTATCACG -3

Reverse 5'-CCTAGGAGAATTCGTCCCATTCCCCGATGAACATGCGTGG-3'

The amplified fragments were cloned into a pUAST vector in EcoRI and KpnI-XbaI restriction sites respectively. For the HA-tagged form an oligo that contains the HA-tag was additionaly cloned in EcoRI-XbaI.

To generate the UAS-CG13183 trangene, genomic DNA was used to perform a PCR using the following primers:

Forward 5'- GGTACCCAAGCTTTGATGATTTCTCGCCGCAAGATCATA -3'

Reverse 5'- TCTAGATCATTCATGTGCGCTGATCAGACTACGTG -5'

The amplified fragment was cloned into a pUAST vector in EcoRI restriction sites.

To generate the *UASkkvGFP* construct, two point mutations present in the open reading frame of the full-length kkv cDNA RE32455 (available from DGRC) were repaired by site directed mutagenesis (Strategene). Next, the cDNA was cloned into the pCR8 vector (Invitrogen). The resulting cDNA was then cloned into the pUASGFP vector pTGW (DGRC) allowing an in frame fusion of the *kkv* sequence to the 3’ end of the GFP sequence.

DNA were then injected in *w^1118^* embryos following standard procedures.

**Generation of CG13188 and CG13183 antibodies**

Polyclonal antibodies against CG13188 were generated using a 1542 bp fragment that encodes the N-terminal region of CG13188-RB. The fragment was amplified by PCR using the following primers with restriction sites:

NdeI: CATATGGTGTCGCGTCGAAAAATT

XhoI: CTCGAGTCAGTCCCATTCCCCGAT

To generate an antibody against CG13183 the following primers were used to amplify a fragment of 516 pb.

NcoI: AGCCAAGTGGGAT

HindIII: TCATGTGCCGTGA

The amplified fragments were cloned into the expression vector pET28a (Novagen) or pROX, which carry an N-terminal His tag. The resulting positive clones were used to transform BL21 (DE3) cells (Novagen) for protein expression. Cells were induced with 1 mM IPTG and proteins were expressed at 37ºC during 2 hours. The positive clones were selected and the recombinant proteins (63 KDa for CG13188 and 19 KDa for CG13183) fused with a His tag were purified through a column of nickel (Quiagen) in denaturalising conditions (8 M urea).

**Generation of constructs for cell transfection experiments**

Exp and Reb cDNAs were obtained by PCR from RE66796 and RE28239 clones (DGRC, Bloomington, IN) using the following primers:

Exp:

Forward: 5’- CCGGAATTCCGCGATGGTGTCGCGTCGA

Reverse: 5’- CCGCTCGAGGTCCCATTCCCCGATGAAC

Reb:

Forward: 5’- CCGGAATTCAAGATGGGATCTCGCCGCA

Reverse: 5’- AAGGAAAAAAAGCGGCCGCTTCATGTGCGCTGATCAG

Kkv cDNA was obtained by PCR from genomic DNA of transgenic UAS-GFPkkv flies using the following primers:

Kkv:

Forward: 5’- AAGGAAAAAAGCGGCCGCTCGCGATGCGGCATCGCCCGATGGCC

Reverse: 5’- CCGCTCGAGCAGGCGACCTGTGCCATTACCGCCGC

GFP-kkv:

Forward: 5’- CCGCTCGAGACCATGGTGAGCAAGGGC

Reverse: 5’- TCCGGGCCCTCACAGGCGACCTGTGCCATT

**Quantitative Real Time PCR**

The following primers were used:

CG13188:

Forward: 5’- ATTGGACTGTGCGGCTTCG

Reverse: 5’- GGATGAGGATGTTCCCAGCG

CG13167:

Forward: 5’-CCTGCCCATCTTTCCATCCC

Reverse: 5’- TTCCGCTTGAGGAGACCCAC

**SUPPLEMENTAL INFORMATION**

Clustal O-alignment of CG13188 (short and long isoforms) and CG13183 with homologous sequences from insects and non-insects (Homo sapiens, Hsap and C. elegans, Cele). Sequences were retrieved by Blast searches at NCBI (Hsap, Agam, Cele), BeeBase (Amel) and BeetleBase (Tcas). The sequence in the grey box represents the MH2 domain. Please note that the MH2 domain of the human protein (Hsap) is rather C-terminal.

Underneath the alignment symbols represent conserved (*) or similar amino acids (colon [:] strong similar properties, period [.] weak similar properties) according to the guidelines for Clustal Omega at http://www.ebi.ac.uk/Tools/msa/clustalo/help/.

CLUSTAL O(1.2.1) multiple sequence alignment

Hsap MPLPEEQYKVGEPTGDWSLDGRLQVSHRKGLPHVIYCRLWRWPDLHSHHELRAMELCEFA

CeleCG13188 ------------------------------------------------------------

DmelCG13183 ------------------------------------------------------------

AmelCG13188 ------------------------------------------------------------

TcasCG13188 ------------------------------------------------------------

AgamCG13188 ------------------------------------------------------------

DmelCG13188short ------------------------------------------------------------

DmelCG13188long ------------------------------------------------------------

Hsap FNMKKDEVCVNPYHYQRVETPVLPPVLVPRHTEIPAEFPPLDDYSHSIPENTNFPAGIEP CeleCG13188 -----------------------MENGVSRSSLVH------------------------- DmelCG13183 --------------------------MISRRKIISRSLD-----------NLDA------ AmelCG13188 --------------------------MVSRRKILSRSRD-----------NLVES----- TcasCG13188 --------------------------MVSRRRILSRSRD-----------DLHMD----- AgamCG13188 --------------------------MVSRRKILSRSRD-----------DLNLD----- DmelCG13188short --------------------------MVSRRKILSRSRD-----------DLNLD----- DmelCG13188long --------------------------MVSRRKILSRSRD-----------DLNLD-----

: * :

Hsap QSNIPETPPPGYLSEDGETSDHQMNHSMDAGSPNLSPNPMSPAHNN--LDLQPVTYCEPA CeleCG13188 ----------GTQTNPNSDT-------------WYDTDAFLLDQVKEVLNKLNEGSIDDE DmelCG13183 ----------IGQEEQEEDV-------------WHDREKLFRDHINEVLSKWE--QIDDE AmelCG13188 --------Q--YEDQDEEDV-------------WYNLDKLYKDHIQEVLDKWN--QIDDE TcasCG13188 --------STFQPPEDEEDV-------------WYQKDKLYKDHIQEVLDKWT--QIDDE AgamCG13188 --------QSYITQEEEEDV-------------WYQKEKLYKEHIQEVLDKWT--QIDDE DmelCG13188short --------QTFTQQEEEEDI-------------WFQKDKLYKEHIQEVLDKWT--QIDDE DmelCG13188long --------QTFTQQEEEEDI-------------WFQKDKLYKEHIQEVLDKWT--QIDDE

: . . : : : : *. :

Hsap FWCSISYYELNQRVGETFHASQPSMTVDGFTDPSNSERFCLGLLSNVNRNAAVELTRRHI CeleCG13188 IWGKIVIMERCKRVAKAYL-RKTTVIIDGSEDEFDGKTLGFNHFENPTRDDHTKEIRAKI DmelCG13183 IWAKIIVFEKNRRVAKAYA-RSSVITINGSKNGFDGVRIGLNGFDNPMRDAETKVIKKSI AmelCG13188 IWAKVIVFERNRRVAKAYA-RAPVLTINGSNDGFDGFRIGLCGFENPMRDPKTEEAKKLI TcasCG13188 IWAKVIVLERNRRVAKAYA-RAPVLTVNGSDDGFDGFRIGLCGFDNPMRDQKTEEFKRHI AgamCG13188 IWAKVIVFERNRRVAKAYA-RAPVLTINGSDDGFDGMRIGLCGFDNPMRDHKTEEVKRHV DmelCG13188short IWAKVIVFERNRRVAKAYA-RAPVLTINGSDDGFDGMRIGLCGFDNPMRDQKTDEMKRVI DmelCG13188long IWAKVIVFERNRRVAKAYA-RAPVLTINGSDDGFDGMRIGLCGFDNPMRDQKTDEMKRVI :* .: * :**.::: : ::* : :. : : :.* *: .. : :

Hsap GRGVRLYY-IGGEVFAECLSDSAIFVQSPNC----NQRYGWHP------ATVCKIPPGCN CeleCG13188 ADGVILKMDYQGNIKGMARGATPIVCQGWK--EPRNNCISDRLVRLHGKLNHGAMEDEKA DmelCG13183 GDGFKIKMDDTGNIFIKRYGKSSIYVNSTSQ-GNEETVIGGDII----QMPQMSLTAATS AmelCG13188 NQGVKIKMDEQGNILIKRLCKNNVYIKPTS---QEDNAIGAEIA----RNSQGALEHEKP TcasCG13188 GHGVKIKMDDAGNILIKRVSKCNVYIKNTGQ--DDENAIGNEIL----KLPNCALEPEKP AgamCG13188 GQGVKIKMDDAGNILIRRYSKSNVYVKSTANQPNEETAIGADIL----KLPGQAIESEKI DmelCG13188short GQGVKIKMDDAGNILIRRYAKSNVYVKSTASSPNEETSIGAEIL----KLPNQALESEKI DmelCG13188long GQGVKIKMDDAGNILIRRYAKSNVYVKSTASSPNEETSIGAEIL----KLPNQALESEKI

*. : *:: : : : . :

Hsap LKIFNNQEFAALLAQSVNQGFEAVYQLTRMCTIRMSFVKGWGAEYRRQTVTSTPCWIELH CeleCG13188 YKVFDMRKFKHSLERELHDGTPDARNLLLKTCMRVALVKDG------ADMNRTPCWFAIV DmelCG13183 ARLFDMKKFQTNINREFARTYPERGRLERQCLSAVSLVKSN------NNLINSPVWILVV AmelCG13188 GKVFDMSKFQTNLSRETRRAYPDRRRLEMQCLSAIVFVRTE------LDLLQCPVWVLIV TcasCG13188 VKLFDMKKFQSNVNRELRRAYPDRRRLECQCLSAIAFVKSE------PELLECPIWVLII AgamCG13188 VKLFDMKKFQSNVNRELRRAYPDRRRLETQCLSAIAFVKSE------NDILDCPMWVLII DmelCG13188short VKLFDMKKFQSNVNRELRRAYPDRRRLETQCLSAVAFVKSE------NDILECPIWVLIV DmelCG13188long VKLFDMKKFQSNVNRELRRAYPDRRRLETQCLSAVAFVKSE------NDILECPIWVLIV

::*: :* : :. .* : :*: : * *. :

Hsap LNGPLQWLDKVLTQMGS----------PSIRCSSVS------------------------ CeleCG13188 NLVALDMIKEKIPLIKSLLNVYGL---PSVTSP--SPMS--GPD------NSGFNAQTLT DmelCG13183 NVVAMDMLRSRLQTIPKSLD--AMGMRVPLTNSSEDPYSTIESQVGLIYPTPAASDDSQ- AmelCG13188 NVVGLDMLKSKLPPVLALQRPVDIKNRPRIPIPDEDPYSIAGVSSS-----IGVSESFQ- TcasCG13188 NVVAMDMLKSKLPPVQR--V-MDIKNRPRIPIPDEDPYSVAGNGS-GSSGSSGFA----- AgamCG13188 NVVAMDMLKSKLPPVQR--P-VDIKNRPRIPIPDEDPYSVAGNGG-GSSGSSGFGAS--- DmelCG13188short NVVAMDMLKSKLPPVQR--PIVDIKNRPRIPIPDEDPYSVAGNGSGGSSGSSGFGSGHQA DmelCG13188long NVVAMDMLKSKLPPVQR--PIVDIKNRPRIPIPDEDPYSVAGNGSGGSSGSSGFGSGHQA

. .. ..:: : . : : : *.

Hsap ------------------------------------------------------------ CeleCG13188 QIIA-----AAVTQANKGTPT-------------------------PTRNNNNNNQNLTI DmelCG13183 --------------------------NSQHSSNSSKGRRS-----IFSAAFLNEGP-Y-V AmelCG13188 ------------------QDRDTRDQIYIQSMN-SRQKRTEKPPKLPPRENLYSHD---I TcasCG13188 ---------------GAGSVAATREQLLLQSQQH-AQRRSEKPPKLPPRENMYPHD---I AgamCG13188 ---------------GSGLVAASREHLMQPAQPMGGQRRSEKPPKLPPRDNLYATH-E-I DmelCG13188short QLHPQQQLGKHLNGNGGGHVAATREQLLLQTQQL-AHKRSEKPPKLPPRDNIYSHD-L-I DmelCG13188long QLHPQQQLGKHLNGNGGGHVAATREQLLLQTQQL-AHKRSEKPPKLPPRDNIYSHD-L-I

Hsap ------------------------------------------------------------ CeleCG13188 SPEQLAQIASTISIAQ--TNTLRTADLVKKERPKKPYHCSTSSLDSSGEDSSGRRSEAST DmelCG13183 PKPDYESGGSLTPIYA-DKKRQKSSGSQQRKKQDDPYYCGLLARIPNFIKSS-KQPCPGG AmelCG13188 PKPDYDDIEDDYARKSIITSEDKRNKHDDKKKYDDPYYCGLRARVPNFVKMA-KSNKISS TcasCG13188 PKPDYDDHEHDNSRLKPFPSSRGKEKSKDSKKYDDPYYCGLRARVPNFVAKS-KAKESV- AgamCG13188 GKPDYDDIEDENRVKL---PRGKSDKGKDNKKYDDPYYCGLRARVPNFVKSS-SKSSKE- DmelCG13188short PKPDYDDIDLETRIKL---SRGKSDKGKDNKKYDDPYYCGLRARVPNFVKSG-KPIPAQV DmelCG13188long PKPDYDDIDLETRIKL---SRGKSDKGKDNKKYDDPYYCGLRARVPNFVKSG-KPIPAQV

Hsap ------------------------------------------------------------ CeleCG13188 AIKSRAKRWEQVQKINEIEKYTESPAVQMLDKPRAGGRAHRPLLQDV-I---FTRSHSIT DmelCG13183 ASK--PKKEPKI-----------SRKISAQHQ-QQQF------LQPAQPIPIATFHHSYF AmelCG13188 RGYSRPTQVPVYT-GTGYT----NSQSSKI------------------------------ TcasCG13188 ----------------------SSKRFSISQQ-A------PPPL------PVMHQ----- AgamCG13188 --NNNNGPNAAVG-VGGSKETVASKRLSIAHM-Q-----HPASLQS------LHQLHQMH DmelCG13188short AKDQRDGNGNNIG-NGGISTL-SQKKTSMIHN-HLGG-MHPHHIQQQQLMAAAAAAHAAQ DmelCG13188long AKDQRDGNGNNIG-NGGISTL-SQKKTSMIHN-HLGG-MHPHHIQQQQLMAAAAAAHAAQ

Hsap ------------------------------------------------------------ CeleCG13188 -----SDY----DSNKEHFDSGSWSSTQSRYKDGRSTSSSV-VSEHELAALARDELKE-- DmelCG13183 PYKLYPPQHRLSQSQMSLWDARSLISAHE------------------------------- AmelCG13188 -------YGHLGNRPPIMYHARSFENSD--SRNESPYNHIYGR----FPIPTRGVIPP-- TcasCG13188 ----QHVNSHHNMHQHSMWHTRSFESGIDSDVVESPYNQIYGR----LPIPTRAYIPS-- AgamCG13188 PH--HNLMAAHQHHQQLMWHARSYESGIDTDVVESPYSHMYGR----VPLPTRGYIPA-- DmelCG13188short HH--HAGHQREQQQHHQIWQTRSYESGIDSELVESPYNHIYGRH---LPLPTRGYV---- DmelCG13188long HH--HAGHQREQQQHHQIWQTRSYESGIGIYKQGGPNAAHQSHKSHALQMQMHQQLQMQM

Hsap ------------------------------------------------------------ CeleCG13188 --------TSEIREANAV---------SEDDEPPTNPPSP--APTVNVPTLPRKDYNQS- DmelCG13183 ------------------------------------------------------------ AmelCG13188 -----TPRAMYIGEWD-------------------------------------------- TcasCG13188 -----QSRTMYIGEWD-------------------------------------------- AgamCG13188 ------PRAMYIGEWD-------------------------------------------- DmelCG13188short -----PTPRMFIGEWD-------------------------------------------- DmelCG13188long QHQQQQQPQSLPTDLTATTATATTKTQSTANQARSHQPHPHLHPHSNPHAHPHHRLHRHV

Hsap ------------------------------------------------------------ CeleCG13188 -------PAHTPEHVCPLPRSFSSTPAAEQFLPTLEGAPVWTPSAVS------------- DmelCG13183 ------------------------------------------------------------ AmelCG13188 ------------------------------------------------------------ TcasCG13188 ------------------------------------------------------------ AgamCG13188 ------------------------------------------------------------ DmelCG13188short ------------------------------------------------------------ DmelCG13188long HVRCERHAHLQQQHSAPLQS--------VQHQPQQQQQPYYDNLEDSVARRVTRRHSTRR
